# The relationship between obstructive sleep apnea and asthma severity and vice versa: a systematic review and meta-analysis

**DOI:** 10.1186/s40001-023-01097-4

**Published:** 2023-03-30

**Authors:** Donghao Wang, Yanyan Zhou, Riken Chen, Xiangxia Zeng, Sun Zhang, Xiaofen Su, Yateng Luo, Yongkang Tang, Shiwei Li, Zhiyang Zhuang, Dongxing Zhao, Yingying Ren, Nuofu Zhang

**Affiliations:** 1 Sleep Medicine Center, State Key Laboratory of Respiratory Disease, National Clinical Research Center for Respiratory Disease, Guangzhou Institute of Respiratory Health, The First Affiliated Hospital of Guangzhou Medical University, Guangzhou, 510120 Guangdong People’s Republic of China; 2 Department of Respiratory and Critical Care Medicine, The Second Affiliated Hospital of Guangdong Medical University, Zhanjiang, People’s Republic of China; 3 Medical Records Management Department, The First Affiliated Hospital of Guangzhou Medical University, Guangzhou, 510120 Guangdong People’s Republic of China

**Keywords:** Obstructive sleep apnea, Asthma, Lung function, Polysomnography, Daytime sleepiness

## Abstract

**Background:**

There is a great association between the prevalence of obstructive sleep apnea (OSA) and asthma. Nonetheless, whether OSA impacts lung function, symptoms, and control in asthma and whether asthma increases the respiratory events in OSA are unknown. This meta-analysis aimed to examine the relationship between obstructive sleep apnea and asthma severity and vice versa.

**Methods:**

We carried out a systematic search of PubMed, EMBASE, and Scopus from inception to September 2022. Primary outcomes were lung function, parameters of polysomnography, the risk of OSA in more severe or difficult-to-control asthmatic patients, and the risk of asthma in patients with more severe OSA. Heterogeneity was examined with the *Q* test and I^2^ statistics. We also performed subgroup analysis, Meta-regression, and Egger’s test for bias analysis.

**Results:**

34 studies with 27,912 subjects were totally included. The results showed that the comorbidity of OSA aggravated lung function in asthmatic patients with a consequent decreased forced expiratory volume in one second %predicted (%FEV1) and the effect was particularly evident in children. %FEV1 tended to decrease in adult asthma patients complicated with OSA, but did not reach statistical significance. Interestingly, the risk of asthma seemed to be slightly lower in patients with more severe OSA (OR = 0.87, 95%CI 0.763–0.998). Asthma had no significant effect on polysomnography, but increased daytime sleepiness assessed by the Epworth Sleepiness Scale in OSA patients (WMD = 0.60, 95%CI 0.16–1.04). More severe asthma or difficult-to-control asthma was independently associated with OSA (odds ratio (OR) = 4.36, 95%CI 2.49–7.64).

**Conclusion:**

OSA was associated with more severe or difficult-to-control asthma with decreased %FEV_1_ in children. The effect of OSA on lung function in adult patients should be further confirmed. Asthma increased daytime sleepiness in OSA patients. More studies are warranted to investigate the effect of asthma on OSA severity and the impact of different OSA severity on the prevalence of asthma. It is strongly recommended that people with moderate-to-severe or difficult-to-control asthma screen for OSA and get the appropriate treatment.

**Supplementary Information:**

The online version contains supplementary material available at 10.1186/s40001-023-01097-4.

## Introduction

The prevalence of obstructive sleep apnea (OSA) and asthma is increasing. Nevertheless, OSA and asthma have adverse effects on each other with distinct interactive mechanisms under the upper and lower airway pathologies, in addition to shared comorbidities such as obesity, rhinitis, and gastro-oesophageal reflux [1]. The prevalence of OSA among asthmatic populations has been reported to be 38 to 70%; one of the main reasons is structural and collapsibility changes in the upper airway during sleep related to the use of inhaled corticosteroids (ICS), oral corticosteroids, and systemic corticosteroids [1, 2]. Conversely, 35.1% of clinical populations with OSA report physician-diagnosed asthma, likely due to chronic intermittent hypoxia with consequent inflammation and/or remodeling of the lower airways [3, 4].

A large retrospective cohort study of patients with OSA showed significantly higher rates of all-cause readmission compared with those without OSA (hazard rate = 1.56; 95% confidence interval 1.50–1.62) [5]. Comorbidity with OSA has a negative effect on the prognosis of asthma. As well as being an important test in the diagnosis of asthma, greater variability in lung function is associated with poorly controlled asthma [6]. Nonetheless, the link between OSA and lung function and asthma control is controversial [7–26]. Although asthma makes a significant contribution to the risk of OSA, it is unclear if asthma aggravates OSA severity with increased respiratory events and more severe hypoxia [27–40]. A meta-analysis to evaluate the relationship between OSA and asthma severity and vice versa is warranted. This review will focus on the independent interaction of OSA and asthma based on a meta-analysis.

## Methods

We conducted a meta-analysis according to PRISMA statement guidelines [41]. The first part, (Part A), examined the effect of OSA on lung function and the risk of asthma in patients with different OSA severity. Part B examined the effect of asthma on PSG and Epworth Sleepiness Scale (ESS), as well as the risk of asthma in patients with different asthma severity (or control status). This meta-analysis has been registered in the PROSPERO database (CRD42021283829).

### Search strategy

**Fig. 1:**
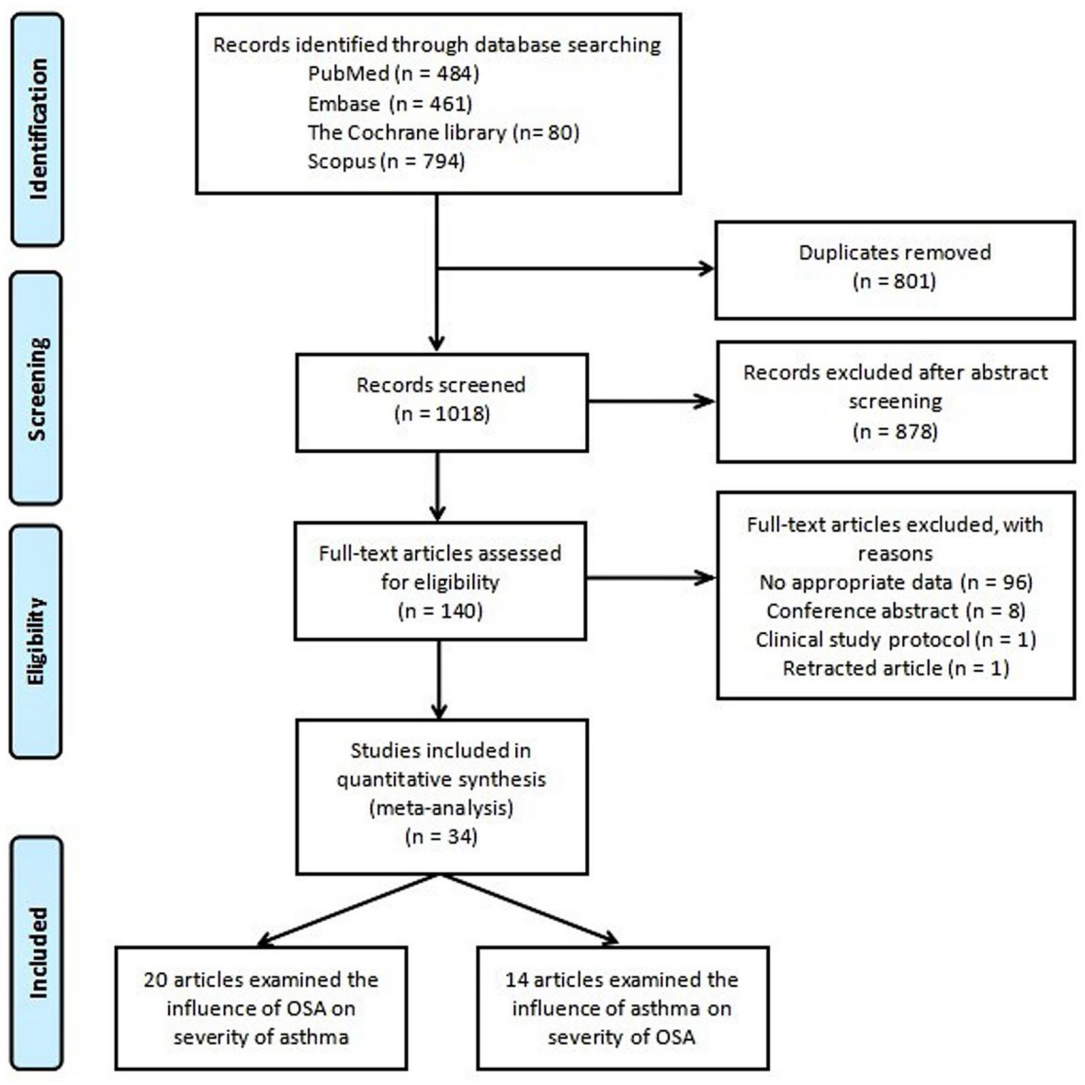
The flow diagram of identifying studies through systemic search in multiple databases. OSA: obstructive sleep apnea

We carried out a systematic search through PubMed, Medline, EMBASE, and Scopus, ClinicalTrials (all searched from inception to September 2022). Index terms such as medical subject headings and free text were utilized to capture a broad range of literature. Index terms were limited to those identified in the title, abstract, and keywords. The detailed strategy listing all search terms used and how they were combined is shown in the Additional file 1: Tables S1–S3) and the selection process is shown in Fig. 1.

### Selection criteria

Two researchers independently assessed the articles for eligibility for inclusion. Any disagreements were resolved through discussion, and the senior author was available to arbitrate if necessary.

The inclusion criteria for Part A were as follows: (1) children or adult patients diagnosed with asthma according to accepted guidelines or criterion; (2) cross-sectional or cohort study including OSA and non-OSA groups in asthmatic patients; (3) at least one of the following outcome measurements included: forced expiratory volume in one second %predicted (%FEV_1_), forced vital capacity % predicted (FVC%), forced expiratory volume in one second/forced vital capacity (FEV_1_/FVC), forced expiratory flow 25–75% of VC (FEF_25-75_%), asthma control test (ACT); (4) sufficient data for calculating odds ratio (OR) with 95% confidence interval (CI) of the risk of asthma in OSA with varying severity; (5) English language.

The inclusion criteria for Part B were: (1) children or adult patients diagnosed with OSA by polysomnography (PSG); (2) cross-sectional or cohort study including asthma and non-asthma groups in OSA patients; (3) at least one of the following outcome measurements included: apnea/hypopnea index (AHI), lowest peripheral oxygen saturation (LSpO_2_), oxygen desaturation index (ODI), arousal index (ArI), Epworth Sleepiness Scale, percent sleeping time in which oxygen saturation was below 90% (T90%); (4) sufficient data for calculating odds ratio (OR) with 95% confidence interval (CI) of the risk of OSA in asthma with varying severity; and (5) English language.

### Data extraction

Information was independently extracted from each study by two authors. Tables from baseline included the following variables: first author, publication year, research site (country), gender (male%), mean age and body mass index (BMI) of the study population, study design, smoking condition, severity of OSA or asthma, study population, and quality of the study. The definition of OSA severity was based on the American Academy of Sleep Medicine. And the definition of asthma severity was based on Global Initiative for Asthma (GINA), including moderate-to-severe asthma, uncontrolled asthma, and difficult-to-treat asthma. If the original article did not define asthma severity according to GINA, the characteristics of the asthmatic population were recorded. Other specific variables included the number of asthma patients with and without OSA (Table 1), number of asthma patients graded by severity and OSA patients in each group (Table 2), number of OSA patients with and without asthma (Table 3), number of OSA patients graded by disease severity and patients with asthma in each group (Table 4), subgroup meta-analysis of main outcomes in patients with asthma in OSA group and non-OSA group (Table 5), and subgroup meta-analysis of main outcomes in patients with OSA in asthma group and non-asthma group (Table 6). In forest plot for the subgroup of OSA severity and asthma severity, we macroscopically focused more on the comparison of more severe OSA or asthma with controls, regardless of the specific degree of OSA severity, and the asthma severity or control status.

**Table 1:** Main characteristics of included studies investigating the effect of OSA on lung function and ACT in asthma patients

Author, year, Country	Population with/without OSA	Age^Φ^ (years)	BMI^Φ^ (kg/cm2)	Male gender (%)	Smoking condition (N or packs/year)	Study design, Quality of study	Severity of asthma	Severity of OSA	Outcomes	Results and implications
Ciftci et al. 2005 [7] Turkey	N = 22/16	44.7 (8.0)	33.6 (6.5)	73.7	N/A	Cohort study, NOS = 7	Patients who had nighttime symptoms and habitual snoring under optimal medication	AHI ≥ 5/h	%FEV_1,_ FVC%, FEV_1_/FVC, FEF_25–75_%	OSA may be a responsible disease for nocturnal symptoms
Kheirandish-Gozal et al. 2011 [8] USA	N = 58/34	6.6 (1.8)	N/A	53.0	N/A	Cross-sectional, AHRQ = 7	Uncontrolled asthma	AHI ≥ 5/h	%FEV_1_	The risk of OSA is exceedingly high in poorly controlled asthmatic children. The treatment of OSA appears to be associated with substantial improvements in the severity of the underlying asthmatic condition
Teng et al. 2014 [9] China	N = 28/23	9.4 (1.7)	18.2 (3.5)	51.0	N/A	Cross-sectional, AHRQ = 6	Routine	AHI ≥ 5/h	%FEV_1,_ FVC%, FEV_1_/FVC, FEF_25–75_%	The effect of asthma plus OSA appeared to be sleep disturbance in slow-wave sleep, snoring, respiratory arousal, and leg movement due to respiratory events
Zidan et al. 2015 [10] Egypt	N = 18/12	50.2 (8.4)	27.4 (4.4)	43.3	N/A	Cross-sectional, AHRQ = 5	Routine	AHI ≥ 5/h	%FEV1	A suspicion is warranted for the overlap of OSA and asthma, particularly in patients with severe asthma
Taillé et al. 2016 [11] France	N = 27/28	47.8 (1.7)	28.4 (0.8)	21.8	N/A	Cross-sectional, AHRQ = 7	Uncontrolled asthmatic patients with poor sleep quality, excluding smokers or ex-smokers (> 10 packs/year)	AHI ≥ 5/h	%FEV_1_, FEV_1_/FVC, ACT	Mild OSA in patients with severe asthma is associated with the increased proportion of neutrophils in sputum and changes in airway remodeling
Wang et al. 2017 [12] China	N = 10/67	59.9 (13.1)	26.8 (4.4)	66.2	N = 2/22^§^	Cross-sectional, AHRQ = 6	Routine	AHI ≥ 5/h	%FEV_1_, FEV_1_/FVC	Asthmatic patients with OSA had substantially greater declines in FEV1 than those without OSA. CPAP treatment alleviated the decline of FEV1 in asthma patients with severe OSA
Shaker et al. 2017 [13] Egypt	N = 12/38	44.9 (10.3)	N/A	44.0	Total N = 5	Cohort study, NOS = 5	Asthmatic patients with ESS score ≥ 11	AHI ≥ 5/h	%FEV_1_, FEV_1_/FVC	There is a bidirectional relationship between OSA and asthma with increasing frequency of OSA with the increasing asthma severity
Lu et al. 2017 [14] China	N = 78/45	47.6 (12.1)	26.4 (3.0)	57.7	N = 29/8^§^	Cross-sectional, AHRQ = 7	Patients were not in asthma exacerbation within the past 6 months	AHI ≥ 5/h	%FEV_1_, FVC%, FEV_1_/FVC, ACT	STOP-Bang questionnaire is a preferable sleep questionnaire better than the Berlin questionnaire for detecting moderate and severe OSA in asthmatic patients
Yen et al. 2017 [15] Vietnam	N = 56/29	9.5 (2.1)	17.4 (2.8)	72.8	Second-hand smoker, Total N = 38	Cross-sectional, AHRQ = 7	Routine	Children ≤ 12 years: AHI ≥ 1/h, Children > 12 years: AHI ≥ 5/h	%FEV1, ACT	The presence of allergic rhinitis, snoring, and apnea during sleep in asthmatic children is associated with a higher risk of OSA
NG et al. 2018 [16] China	N = 41/81	50.5 (12.0)	25.9 (4.8)	30.3	OSA group 0.1 (0.6) packs/year^Φ^ Non-OSA group 0.3 (1.5) packs/year^Φ^	Cohort study, NOS = 8	Uncontrolled asthma	AHI ≥ 15/h	%FEV_1_, FVC%, ACT	A high risk of OSA was found among patients with asthma and snoring. CPAP therapy for 3 months did not enhance asthma control but improved daytime sleepiness, quality of life, and vitality
He et al. 2019 [17] USA	N = 41/49	11.2 (3.8)	N/A	57.2	N/A	Cross-sectional, AHRQ = 7	Moderate to severe persistent asthma	AHI ≥ 5/h	%FEV_1_, FVC%, FEV_1_/FVC, FEF_25-75_%	OSA in children with moderate to severe persistent asthma is associated with a diminished capacity of the lungs to maintain blood gas homeostasis as measured by plant gain and decreased chemoreceptor sensitivity measured by controller gain
Oyama et al. 2020 [18] Japan	N = 21/39	65.0 (13.4)	26.5 (4.8)	20.0	N/A	Cross-sectional, AHRQ = 7	Patients with suspected OSA	AHI ≥ 15/h	%FEV_1_, FEV_1_/FVC	Patients with high AHI tended to require treatment for serious asthma despite having a good respiratory function
Lin et al. 2021 [19] China	N = 93/145	44.4 (7.1)	24.4 (3.8)	53.4	N/A	Cross-sectional, AHRQ = 7	Routine	AHI ≥ 5/h	%FEV_1_, FVC%, FEV_1_/FVC	Allergic rhinitis, BMI, neck circumference, AHI, SaO2, mPAP, and VEGF are risk factors associated with asthma complicated by OSAHS

^Φ^presented as mean (SD); ^§^N = The number of smokers in OSA group/without OSA group; OSA; obstructive sleep apnea, BMI; body mass index, AHI; apnea/hypopnea index, %FEV_1_; forced expiratory volume in one second (%predicted), FVC%; forced vital capacity (%predicted), FEV_1_/FVC; forced expiratory volume in one second/forced vital capacity, FEF_25-75_%; forced expiratory flow (25–75% of VC), NOS; Newcastle–Ottawa Scale, AHRQ; Agency for Healthcare Research and Quality, ACT; asthma control test, CPAP; continuous positive airway pressure, N/A; not available

**Table 2:** Main characteristics of included studies investigating the risk of asthma in patients with varying severity of OSA

Author, year, Country	Population	Co-exist Asthma	Age^Φ^ (years)	BMI^Φ^ (kg/cm^2^)	Male gender (%)	Study design, Quality of study	Severity of asthma	Results and implications
Robichaud-Hallé et al. 2012 [36] Canada	N = 48 (Severe OSA group)	9	55.5	33.9 (7.4)	65	Cross-sectional, AHRQ = 7	Routine	Severe OSA is associated with severe multimorbidity and sub-scores of multimorbidity, including asthma
	N = 36 (Moderate OSA group)	13						
Gutierrez et al. 2013 [37] USA	N = 57 (Moderate/severe OSA group)	24	6.1 (0.3)	20.1 (0.6)	60.3	Cross-sectional, AHRQ = 7	Routine	Asthma is associated with REM-related breathing abnormalities in children with moderate-severe OSA. The link between asthma and REM-related OSA is independent of asthma control and obesity
	N = 84 (Mild OSA group)	38						
Greenberg-Dotan et al. 2014 [30] Israel	N = 593 (Severe OSA group)	50	55.5 (11.1)	N/A	76.5	Cross-sectional, AHRQ = 7	Routine	Patients with asthma and combined COPD/asthma showed no difference in the risks of these co-morbidities between those with and without OSA
	N = 445 (Moderate OSA group)	44						
Pinto et al. 2016 [38] Brazil	N = 50 (Severe OSA group)	4	50.1 (12.9)	29.0 (5.0)	84	Cross-sectional, AHRQ = 7	Routine	In the study, only 4% of patients had asthma associated with OSA, although it has not presented expressive values; only patients with severe apnea had associated asthma
	N = 34 (Moderate OSA group)	0						
Tamanyan et al. 2016 [39] Australia	N = 75 (Moderate/severe OSA group)	19	6.8 (3.2)	N/A	58.9	Cross-sectional, AHRQ = 7	Not mentioned	In addition to ethnicity (non-Caucasian) and paternal smoking, obesity and a history of asthma and/or allergic rhinitis were not found to be associated with the severity of sleep-disordered breathing
	N = 76 (Mild OSA group)	12						
Bonsignore et al. 2018 [35] Italy	N = 5578 (Severe OSA group)	241	53.5 (11.9)	32.9 (7.8)	74.8	Cross-sectional, AHRQ = 8	Routine	The overall risk of physician-diagnosed asthma was around 5%, with the expected higher risk in women compared with men. The risk of asthma was highest in OSA-free subjects, with a tendency to progressively decrease with increasing OSA severity
	N = 7019 (Mild/moderate OSA group)	341						
Tveit et al. 2018 [40] Norway	N = 613 (Moderate/severe OSA group)	86	48.6 (17.6)	N/A	70.6	Cross-sectional, AHRQ = 7	Routine	There was no association between OSA severity and stroke, COPD, and asthma in the patient group
	N = 558 (Mild OSA group)	93						

^Φ^presented as mean (SD); OSA; obstructive sleep apnea. BMI; body mass index. AHI; apnea/hypopnea index. NOS; Newcastle–Ottawa Scale. AHRQ; Agency for Healthcare Research and Quality. N/A; not available

**Table 3:** Main characteristics of included studies investigating the effect of asthma on PSG and ESS in OSA patients

Author, year, Country	Population with/without asthma	Age^Φ^ (years)	BMI^Φ^ (kg/cm^2^)	Male gender (%)	Smoking condition (N or pack year)	Study design, Quality of study	Severity of OSA	Severity of asthma	Outcomes	Results and implications
Bonay et al. 2003 [27] France	N = 15/22	54.0 (11.7)	36.6 (8.1)	64.9	Asthma group 28 (27) pack year^Φ^ Non-asthma group 9 (17) pack year^Φ^	Cross-sectional, AHRQ = 5	AHI ≥ 15/h	Routine	AHI	Lung function and bronchial responsiveness may be impaired by long-term treatment of OSA by nCPAP. The impairment is observed only in patients with normal initial lung function
Alharbi et al. 2009 [36] Saudi Arabia	N = 213/393	40.0 (14.5)	36.3 (9.7)	66.7	N/A	Cross-sectional, AHRQ = 6	AHI ≥ 5/h	Routine	AHI, LSpO_2_, ODI, ArI, ESS	There was a high risk of asthma (35.1%) in patients with OSA as compared to the risk of asthma in the general population
Ramagopal et al. 2009 [22] USA	N = 22/28	9.3 (3.4)	26.1(11.0)	64.0	N/A	Cross-sectional, AHRQ = 6	AHI ≥ 1/h	Routine	AHI, LSpO_2_, ArI,	A lifetime history of asthma, extracted from the International Study of Asthma and Allergy in Childhood, was associated with more severe OSA
Greenberg-Dotan et al. 2014 [30] Israel	N = 96/100	56.6 (11.1)	32.5 (7.0)	N/A	Asthma group 21 (16) pack year^Φ^ Non-asthma group 29 (13) pack year^Φ^	Cross-sectional, AHRQ = 7	AHI ≥ 5/h	Routine	AHI, ArI, T90%	Patients with asthma and combined COPD/asthma showed no difference in the risks of these co-morbidities between those with and without OSA
Teng et al. 2014 [9] China	N = 28/28	9.5 (1.2)	17.9 (2.9)	62.5	N/A	Cross-sectional, AHRQ = 6	AHI ≥ 5/h	Routine	AHI, ArI, T90%	The effect of asthma plus OSA appeared to be sleep disturbance in slow-wave sleep, snoring, respiratory arousal, and leg movement due to respiratory events
Zaffanello et al. 2017 [31] Italy	N = 28/98	7.8 (4.1)	21.3 (7.1)	56.3	N/A	Cross-sectional, AHRQ = 8	Not mentioned	Routine	LSpO_2_, ODI	Children with recurrent wheeze/asthma showed an increased number of central sleep apnea compared to unaffected children
Bonsignore et a. 2018 [32] Italy	N = 241/5337	54.0 (12.2)	33.9 (8.4)	80.9	N/A	Cross-sectional, AHRQ = 8	AHI ≥ 30/h	Routine	AHI, LSpO_2_, ODI, ESS	The overall risk of physician-diagnosed asthma was around 5%, with the expected higher risk in women compared with men. The risk of asthma was highest in OSA-free subjects, with a tendency to progressively decrease with increasing OSA severity
Sundbom et al. 2018 [33] Sweden	N = 15/109	55.6 (9.7)	28.8 (8.5)	0	N = 4/38^δ^	Cross-sectional, AHRQ = 5	AHI ≥ 15/h	Routine	AHI, ODI, T90%	Co-exist asthma and OSA are associated with poorer sleep quality and more profound nocturnal hypoxemia than either of the conditions alone
Shrestha et al. 2019 [34] Curacao	N = 223/2599	66.0 (11.9)	29.0 (4.8)	59.4	Asthma group 1.42 pack year^§^ Non-asthma group 1.88 pack year^§^	Cross-sectional, AHRQ = 7	AHI ≥ 5/h	Routine	AHI, ArI, T90%, ESS	OSA was more severe in a non-asthmatic subgroup, and asthmatics had statistically significant higher ESS scores and sleep latency
Antonaglia et al. 2022 [35] Italy	N = 35/36	62.0 (11.0)	31.2 (14.7)	73.2	N/A	Cross-sectional, AHRQ = 8	AHI ≥ 5/h	Routine	AHI ESS	Asthma may influence the phenotype of OSA by reducing the arousal threshold such that the coexistence of asthma and OSA could be considered a syndrome or a clinical phenotypic trait of OSA

^§^presentsed as median; ^Φ^presented as mean (SD); ^δ^The number of smokers in asthma group/without asthma group; BMI; body mass index, OSA; obstructive sleep apnea, AHI; apnea/hypopnea index, NOS; Newcastle–Ottawa Scale, AHRQ; Agency for Healthcare Research and Quality, LSpO2; lowest peripheral oxygen saturation, ODI; oxygen desaturation index, ArI; arousal index, ESS; Epworth Sleepiness Scale, T90%; percent sleeping time in which oxygen saturation was below 90%, CPAP; continuous positive airway pressure, COPD; chronic obstructive pulmonary disease, N/A; not available

**Table 4:** Main characteristics of included studies investigating the risk of OSA in patients with varying severity of asthma

Author, year, Country	Population	Co-exist OSA	Age^Φ^ (years)	BMI^Φ^ (kg/cm^2^)	Male gender (%)	Study design, Quality of study	Severity of OSA	Results and implications
Brinke et al. 2005 [20] Netherlands	N = 39 (Patients with difficult-to-treat asthma and ≥ 3 severe asthma exacerbations in the 12 months)	5	41.5 (14.1)	N/A	27.0	Cross-sectional, AHRQ = 6	AHI ≥ 5/h	Psychopathology, chronic sinusitis, gastro-oesophageal reflux, (bacterial) respiratory infections, and obstructive sleep apnoea appear to be associated with frequent exacerbations of asthma
	N = 24 (Patients with difficult-to-treat asthma and 1 severe asthma exacerbation in 12 months)	1						
Julien et al. 2009 [21] Canada	N = 26 (Severe asthma group)	23	48.4 (1.9)	27.8 (1.2)	50.0	Cross-sectional, AHRQ = 6	AHI ≥ 15/h	OSA is significantly more prevalent among patients with severe compared with moderate asthma, and more prevalent for both asthma groups than controls without asthma
	N = 26 (Moderate asthma group)	15						
Teodorescu et al. 2012 [22] USA	N = 283 (Patients with persistent nighttime asthma symptoms)	181	47.0 (14.0)	29.0 (7.0)	33.2	Cross-sectional, AHRQ = 8	AHI ≥ 5/h	OSA is associated with persistent daytime asthma symptoms, to an extent that matched or exceeded associations with nighttime asthma symptoms
	N = 469 (Patients without persistent nighttime asthma symptoms)	194						
Byun et al. 2013 [23] Korea	N = 44 (Moderate to severe group)	37	58.8 (12.0)	25.3 (3.8)	58.5	Cross-sectional, AHRQ = 7	AHI ≥ 5/h	Moderate to severe asthma showed a strong correlation with OSA (AHI ≥ 5/h)
	N = 21 (Control group)	3						
Zidan et al. 2015 [10] Egypt	N = 8 (Uncontrolled asthma)	6	50.2 (8.4)	27.4 (4.4)	43.3	Cross-sectional, AHRQ = 5	AHI ≥ 5/h	A suspicion is warranted for the overlap of OSA and asthma, particularly in patients with severe asthma
	N = 15 (Partly controlled asthma)	11						
Wang et al. 2016 [24] China	N = 59 (Patients occurred severe asthma exacerbations over 1 year)	24	46.7 (7.3)	24.1 (4.8)	69.9	Cohort study, NOS = 7	AHI ≥ 5/h	The patients with asthma had a high risk of OSA, which was an important factor associated with severe asthma exacerbations
	N = 87 (Patients did not occur severe asthma exacerbations over 1 year)	4						
Shaker et al. 2017 [13] Egypt	N = 18 (Patients with severe persistent asthma)	10	44.9 (10.3)	N/A	44.0	Cohort study, NOS = 5	AHI ≥ 5/h	There is a bidirectional relationship between OSA and asthma with increasing frequency of OSA with the increasing asthma severity
	N = 21 (Patients with moderate persistent asthma)	2						
Lu et al. 2017 [14] China	N = 8 (Severe asthma group)	6	47.6 (12.1)	26.4 (3.0)	57.7	Cross-sectional, AHRQ = 7	AHI ≥ 5/h	STOP-Bang questionnaire is a preferable sleep questionnaire better than the Berlin questionnaire for detecting moderate and severe OSA in asthmatic patients
	N = 54 (Moderate asthma group)	37						
Yen et al. 2017 [15] Vietnam	N = 44 (Uncontrolled asthma)	30	9.5 (2.1)	17.4 (2.8)	72.8	Cross-sectional, AHRQ = 7	Children ≤ 12 years: AHI ≥ 1/h, children > 12 years: AHI ≥ 5/h	The presence of allergic rhinitis, snoring, and apnea during sleep in asthmatic children is associated with a higher risk of OSA
	N = 25 (Partly controlled asthma)	16						
Megersa et al. 2022 [25] Ethiopia	N = 100 (Patients occurred severe asthma exacerbations over 1 year)	47	44.3 (12.1)	N/A	38.5	Cohort study, NOS = 6	N/A	Upper respiratory tract infection, obstructive sleep apnea, passive smoker, spring season, kitchen smoke, pet ownership, rhinitis, and being jobless were identified as significant determinants of an asthma attack
	N = 200 (Patients did not occur severe asthma exacerbations over 1 year)	27						
Saif M et al. 2022 [26] Oman	N = 163 (Uncontrolled asthma)	69	56.6 (12.4)	40.3 (12.2)	N/A	Cross-sectional, AHRQ = 8	AHI ≥ 5/h	The risk of OSA was high (32.37%) in patients with severe asthma Uncontrolled severe asthma was significantly associated with severe OSA
	N = 135 (Controlled asthma)	32						

^Φ^ presented as mean (SD); BMI; body mass index, OSA; obstructive sleep apnea, AHRQ; Agency for Healthcare Research and Quality, COPD; chronic obstructive pulmonary disease, N/A; not available

**Table 5:** Subgroup meta-analysis of main outcomes in patients with asthma in OSA group and non-OSA group

Outcomes and subgroups	No. of studies	Population	WMD	95%CI	I^2^ (%)	PH	Statistical model	PZ	Between-group *P* value
FEV1 (%)									
All	13	1111	− 2.29	− 4.91, 0.33	75.9	< 0.001	Random	0.086	–
Age									
Children	4	318	− 4.33	− 7.82, − 0.83	48.6	0.120	Random	0.015	0.210
Adult	9	793	− 1.24	− 4.56, 2.08	78.1	< 0.001		0.464	–
Severity of OSA									
AHI ≥ 1 or 5/h	11	1013	− 3.04	− 5.68, − 0.40	77.3	< 0.001	Random	0.024	0.015
AHI ≥ 15/h	2	98	7.69	− 0.54, 15.93	0	0.557		0.067	–
Severity of asthma									
Routine	8	714	− 2.56	− 5.83, 0.70	72.6	0.001	Random	0.124	0.863
Uncontrolled	4	307	− 1.34	− 7.23, 4.54	86.1	< 0.001		0.655	–
FVC (%)	13	1111	0.01	− 1.92, 1.94	0	0.573	Fixed	0.992	–
FEV1/FVC (%)									
All	9	782	− 0.82	− 3.08, 1.44	69.0	0.001	Random	0.477	–
Age									
Children	2	141	− 4.75	− 8.55, − 0.95	0	0.446	Random	0.014	0.035
Adult	7	641	0.04	− 2.28, 2.37	67.3	0.005		0.972	–
Severity of OSA									
AHI ≥ 1 or 5/h	7	684	− 1.65	− 4.03, 0.74	72.0	0.002	Random	0.176	0.017
AHI ≥ 15/h	2	98	4.83	0.05, 9.61	0	0.482		0.047	–
Severity of asthma									
Routine	6	589	− 1.11	− 4.63, 2.41	76.6	0.001	Random	0.536	0.254
Uncontrolled	2	94	1.01	− 0.27, 2.30	0	0.873		0.123	–
FEF_25–75_%	3	179	− 5.15	− 12.72, 2.42	27.0	0.254	Fixed	0.183	–
ACT score	4	385	− 0.37	− 0.74, 0.00	0	0.688	Fixed	0.050	–

WMD; weighted mean difference, CI; confidence interval, PZ; P value for Z test, PH; P value based on Q test for between-study heterogeneity, %FEV1; forced expiratory volume in one second (%predicted), OSA; obstructive sleep apnea, AHI; apnea/hypopnea index, FVC%; forced vital capacity (%predicted), FEF_25-75_%; forced expiratory flow (25–75% of VC), ACT; asthma control test

**Table 6:** Subgroup meta-analysis of main outcomes in patients with OSA in asthma group and non-asthma group

Outcomes and subgroups	No. of studies	Population	WMD	95%CI	I^2^ (%)	PH	Statistical model	PZ	Between-group *P* value
AHI (events/hour)									
All	9	9540	0.47	− 1.59, 2.52	65.9	0.003	Random	0.656	–
Age									
Adult	7	9434	0.72	− 1.95, 3.38	74.2	0.001	Random	0.599	0.670
Children	2	106	− 0.08	− 2.61, 2.44	0	0.703		0.948	–
Severity of OSA									
AHI ≥ 1 or 5/h	5	3801	0.27	− 1.97, 2.50	43.0	0.135	Random	0.686	0.912
AHI ≥ 15/h	3	5739	0.63	− 5.45, 6.71	82.1	0.004		0.839	–
LSpO_2_ (%)	4	6360	− 0.10	− 1.28, 1.09	47.2	0.128	Fixed	0.872	–
ODI (events/hour)	4	6310	− 0.56	− 1.68, 0.56	30.2	0.231	Fixed	0.326	–
ArI (events/hour)	3	3074	− 0.94	− 2.14, 0.26	4.6	0.351	Fixed	0.123	–
T90 (%)	3	3142	0.02	− 0.42, 0.47	0	0.536	Fixed	0.915	–
ESS score	4	9089	0.60	0.16, 1.04	0	0.678	Fixed	0.007	–

WMD; weighted mean difference, CI; confidence interval, PZ; *P* value for *Z* test, PH; P value based on Q test for between-study heterogeneity, AHI; apnea/hypopnea index, LSpO_2_; lowest peripheral oxygen saturation, ODI; oxygen desaturation index, ArI; arousal index, T90%; percent sleeping time in which oxygen saturation was below 90%, ESS; Epworth Sleepiness Scale

### Statistical methods

All analyses were performed using the comprehensive Meta-Analysis Software (Stata 15.0). The random-effects model was conducted if significant heterogeneity was determined (*p* < 0.05); otherwise, the fixed-effects model was applied (*p* ≥ 0.05). We used the I^2^ index to assess heterogeneity. I^2^ ≥ 50 was considered moderate-to-high heterogeneity. Subgroup analyses were conducted to determine the sources of heterogeneity when I^2^ ≥ 50. The pooled weight mean difference (WMD) of each study and 95%CI were used to estimate the following outcomes: %FEV_1_, FVC%, FEV_1_/FVC, FEF_25–75_%, ACT, AHI, LSpO2, ODI, ArI, ESS, and T90%. OR and 95% CI were used to estimate the risk of asthma in OSA with different grades of severity and the risk of OSA in asthma with different degrees of severity. A *P*-value < 0.05 was considered statistically significant.

### Quality assessment

Agency for Healthcare Research and Quality (AHRQ) [42] was used to assess the quality and risk of bias of included cross-sectional studies. The Newcastle–Ottawa scale (NOS) [43] scoring system was used to assess the quality and risk of bias of included longitudinal studies. The publication bias of included studies was assessed by funnel plot, Meta-regression Egger’s test [44]. Sensitivity analyses were conducted to assess the stability of the results.

## Results

### Study selection

According to our research strategy, 1646 articles were retrieved initially and after excluding duplicates, 882 articles remained. After the abstract screening, 752 articles that did not meet the inclusion criterion were excluded. After reading the full text of the remaining 130 articles, a further 99 were excluded: 89 due to a lack of appropriate data, 8 conference abstracts without available data, 1 clinical study protocol, and 1 article that was retracted. 34 eligible studies were included with a total sample size of 27,722 subjects [7–40]. In Part A, 13 studies [7–19] examined the effect of OSA on lung function and ACT in asthma with 1,111 subjects (Table 1), and 7 studies [20–26] examined the relationship between asthma and varying severity of OSA with 15,266 subjects (Table 2). In Part B, 10 studies [9, 22, 25, 27–33] examined the effect of asthma on PSG and ESS in OSA patients with 9,666 subjects (Table 3), and 11 studies [10, 13–15, 34–40] examined the relationship between OSA and varying severity of asthma with 1869 subjects (Table 4). Other characteristics of the included studies are presented in each table.

### The effect of OSA on lung function and ACT in asthmatic patients

In 13 studies (N = 1111) that fulfilled the inclusion criterion, OSA was not significantly associated with aggravation of lung function. The initial meta-analysis indicated that OSA had no influence on %FEV_1_ (pooled WMD = − 2.29, 95%CI − 4.91–0.33, I^2^ = 75.9%), FVC% (pooled WMD = 0.01, 95%CI − 1.92–1.94, I^2^ = 0%), FEV_1_/FVC (pooled WMD = − 0.82, 95%CI − 3.08–1.44, *I*^2^ = 69.0%), or FEF_25–75_% (pooled WMD = − 5.15, 95%CI − 12.72–2.42, *I*^2^ = 27%) (Table 5). The result of ACT scoring revealed that the comorbidity of OSA tended to make asthma more difficult to control (pooled WMD = − 0.37, 95%CI − 0.74–0.00, *P* = 0.05) (Table 5, Additional file 1: Fig. S1). OSA did not significantly affect the result of FEF_25–75_% (pooled WMD = − 5.15, 95%CI − 12.72–2.42, *P* = 0.183) (Additional file 1: Fig. S2). Nonetheless, further analysis suggested that OSA increased fractional exhaled nitric oxide (FeNO) in patients with asthma (pooled WMD = 4.37, 95%CI 0.05–8.69, *P* = 0.047) (Additional file 1: Fig. S3).

**Fig. 2:**
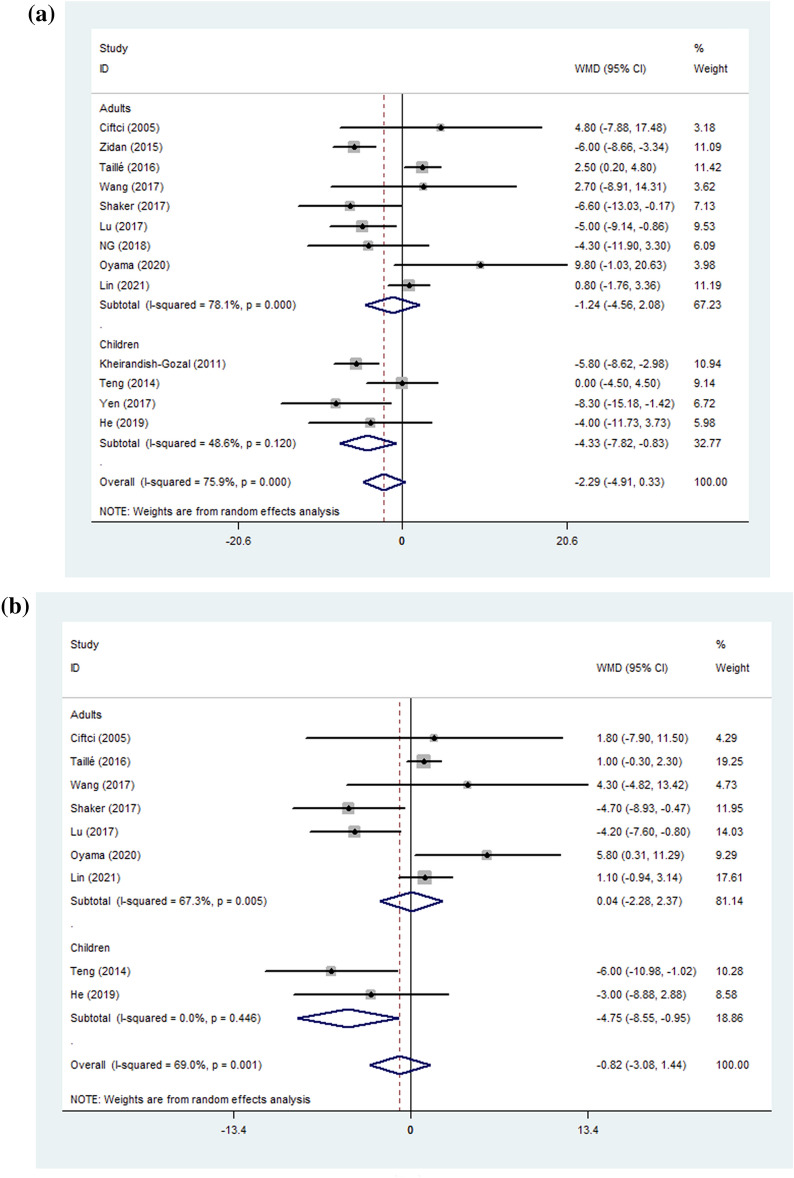
**A** %FEV1 **B**: FEV1/FVC; Forest plot for subgroup analyses by age on the effect of OSA on lung function. %FEV1: forced expiratory volume in one second %predicted; FEV1/FVC: forced expiratory volume in one second/forced vital capacity; WMD: weighted mean difference; CI: confidence interval

**Fig. 3:**
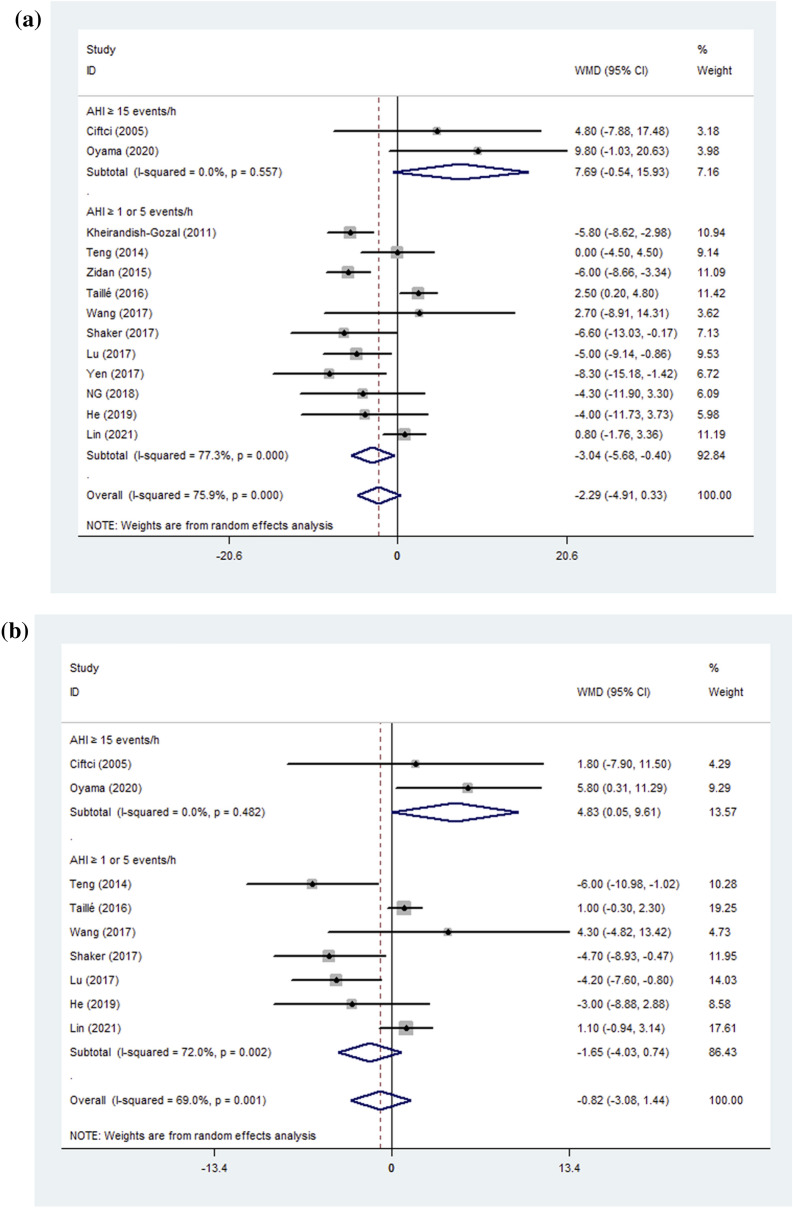
**A** %FEV1; **B** FEV1/FVC; Forest plot for subgroup analyses by OSA severity on the effect of OSA on lung function. %FEV1: forced expiratory volume in one second %predicted; FEV1/FVC: forced expiratory volume in one second/forced vital capacity

The results for %FEV1 and FVC% revealed high heterogeneity so a subgroup analysis was performed to determine the sources of heterogeneity. Analysis after subgrouping subjects by age (children and adults) revealed that %FEV_1_ and FEV_1_/FVC decreased by 4.33 (pooled WMD = − 4.33, 95%CI − 7.82–− 0.83, *P* = 0.015, between-group *P* value = 0.210) (Fig. 2) and 4.75 (pooled WMD = − 4.75, 95%CI − 8.55– − 0.95, *P* = 0.014, between-group *P* value = 0.035) (Fig. 2), respectively, in children with asthma complicated by OSA but no such effect was seen in adults (the heterogeneity evaluated by I^2^ for a subgroup is shown in Table 5). Analysis based on the severity of OSA (AHI ≥ 1 or 5/h, AHI ≥ 15/h) revealed that %FEV_1_ was slightly decreased in patients with asthma complicated by OSA (AHI ≥ 1 or 5/h) (pooled WMD = − 3.04, 95%CI − 5.68–− 0.40, *P* = 0.024, between-group *P* value = 0.015) (Fig. 3) but tended to increase (pooled WMD = 7.69, 95%CI − 0.54–15.93, *P* = 0.067) in patients with AHI ≥ 15/h. Interestingly, FEV_1_/FVC significantly improved (pooled WMD = 4.83, 95%CI 0.05–9.61, *P* = 0.047, between-group *P* value = 0.017) in patients with AHI ≥ 15/h, but was not significantly affected when OSA complicated with asthma (AHI ≥ 1 or 5/h) (Fig. 3). We also conducted a subgroup analysis based on the severity of asthma. There were no statistically significant differences when patients with asthma but no additional conditions (routine group) were compared with an uncontrolled group (defined according to the criterion of GINA) (Table 5).

Since the pooled results of %FEV1 and FEV1/FVC in adult patients with asthma were inconsistent with high heterogeneity, more subgroup analyses were performed to address this. When evaluating %FEV1 and FEV1/FVC, the results of subgroup analysis based on OSA severity were similar to those of the all age group, containing 2 same articles in a group of AHI ≥ 15/h [7, 18]. However, the decline in %FEV1 (pooled WMD = − 2.34, 95%CI − 5.78–1.10, *P* = 0.182, *I*^2^ = 81.1%, between-group *P* value = 0.028) and FEV1/FVC (pooled WMD = − 0.79, 95%CI − 3.26–0.79, *P* = 0.535, *I*^2^ = 72.6%, between-group *P* value = 0.041) showed no significant differences in asthmatic patients with AHI  > 5/h (Additional file 1: Table S4). There were no significant differences among subgroups regarding severity of asthma, age, male%, and BMI, with remaining high heterogeneity (all between-group *P* values > 0.05, Additional file 1: Table S4).

### The risk of asthma in patients with different OSA severity

**Fig. 4:**
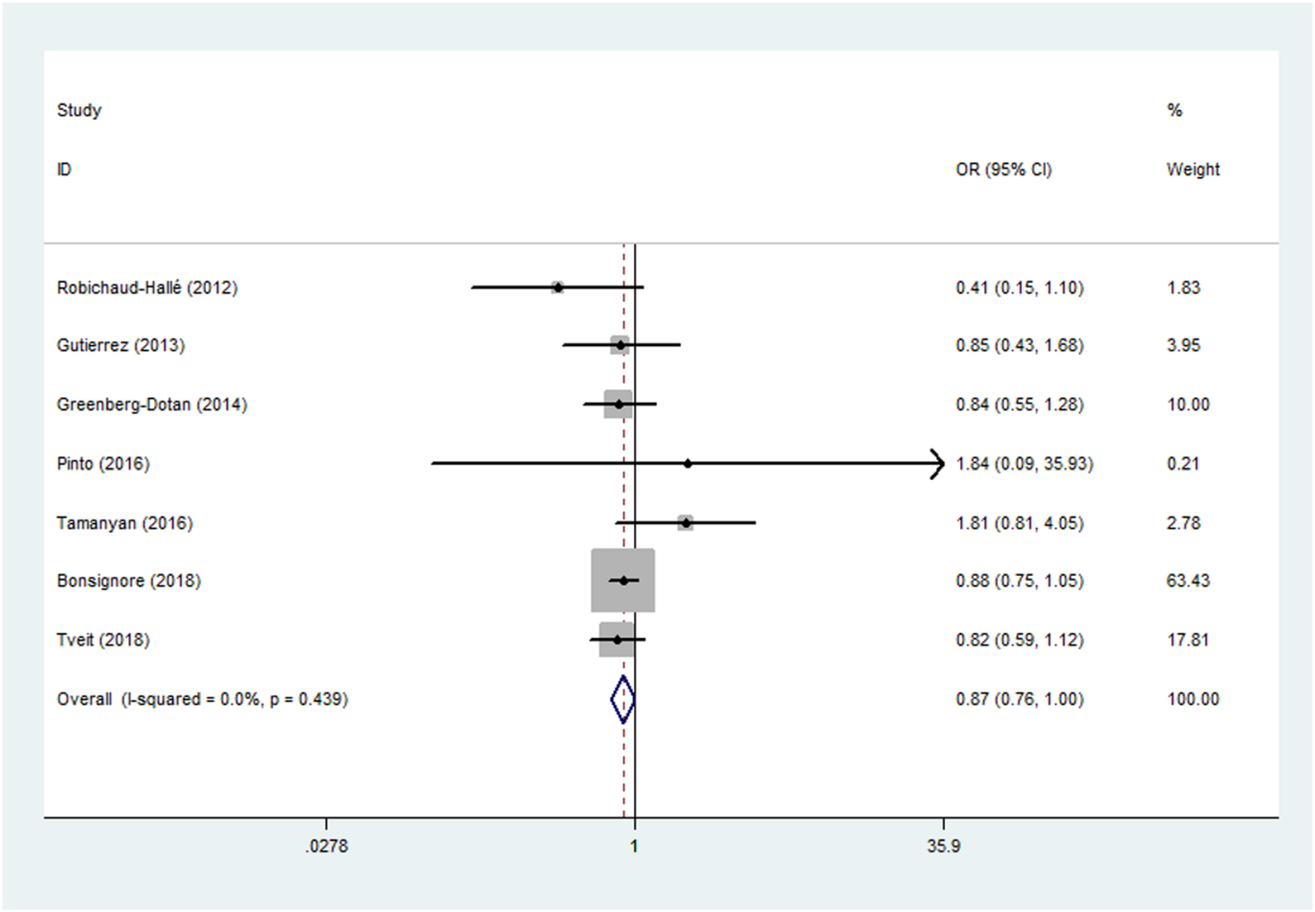
Forest plot for Meta-analysis of odds ratio of OSA in patients with varying severity of asthma. OSA: obstructive sleep apnea; OR: odds ratio

When exploring the association of asthma with OSA severity, we found that the risk of asthma was lower in patients with more severe OSA with low heterogeneity (pooled OR = 0.87, 95%CI 0.763 to 0.998, *I*^2^ = 0%, *P* = 0.047) in a large number of subjects (N = 15,266). The result suggested that people with more severe OSA are less likely to be complicated with asthma. The characteristics of included OSA patients and the forest plot are shown in Table 2 and Fig. 4, respectively.

### The effect of asthma on lung function and the ESS score in OSA patients

A review of 10 studies (N = 9666) that fulfilled the inclusion criterion revealed that asthma had no significant effect on OSA severity when assessed mainly by parameters of PSG (Table 6). There was an insignificant association with AHI (pooled WMD = 0.47, 95%CI − 1.59–2.52, *I*^2^ = 65.9%), LSaO_2_ (pooled WMD = 0.07, 95%CI − 1.28–1.09, *I*^2^ = 47.2%), ODI (pooled WMD = − 0,56, 95%CI − 1.68–0.56, *I*^2^ = 30.2%), ArI (pooled WMD = − 0.94, 95%CI − 2.14–0.26, *I*^2^ = 46%) and T90% (pooled WMD = 0.02, 95%CI − 0.42–0.47, *I*^2^ = 0.0%), regardless of subgroup analyses by age (children and adults) or severity of OSA (AHI ≥ 5/h, AHI ≥ 15/h). In addition, our study suggested that comorbidity of asthma may exacerbate daytime sleepiness assessed by ESS (pooled WMD = 0.60, 95%CI 0.16–1.04, *P* = 0.007) (Additional file 1: Fig. S4).

We also conducted several subgroup analyses based on age, male%, and BMI for adult OSA patients. The results showed that there were no significant differences among subgroups (Additional file 1: Table S5).

### The risk of OSA in patients with different asthma severity

**Fig. 5:**
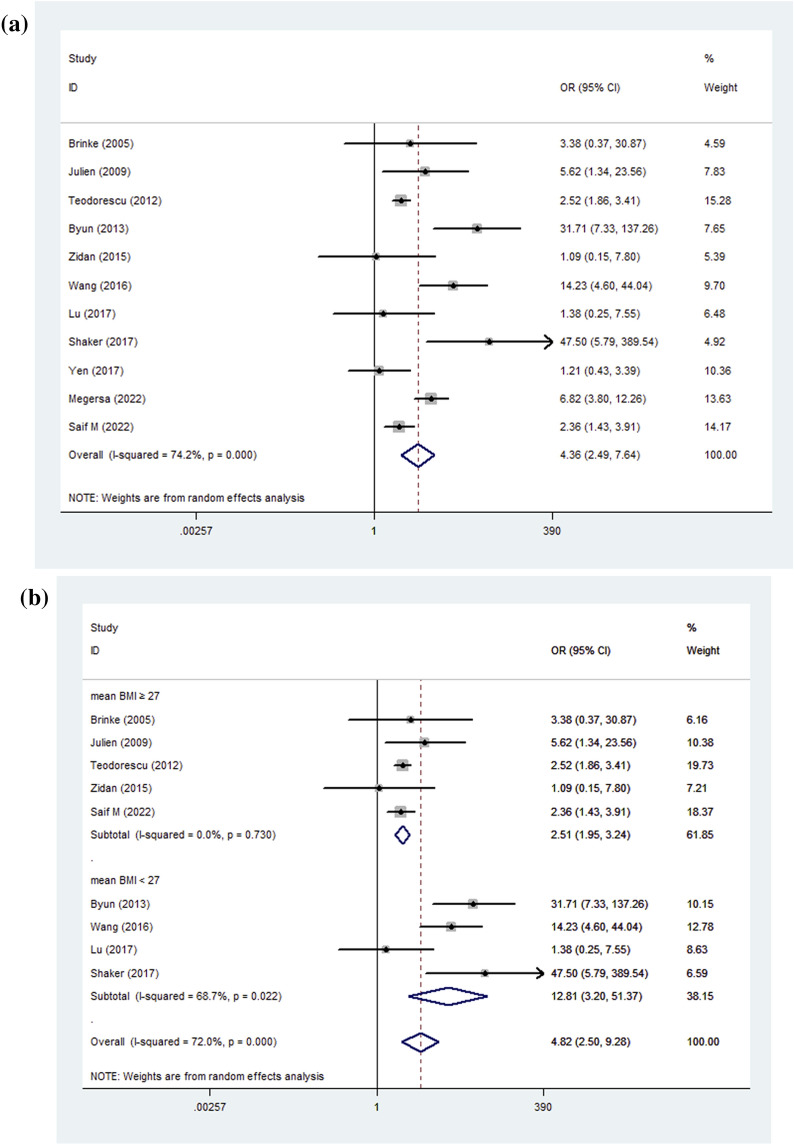
**A** Pooled results; **B** The subgroup of asthmatic patients with BMI < 27; Forest plot for Meta-analysis of odds ratio of asthma in different severity of OSA. OSA: obstructive sleep apnea; BMI: body mass index; OR: odds ratio

The risk of OSA was significantly higher in more severe or more difficult-to-control asthma in 7 of the 11 studies (Total N = 1869). Assessment of asthma severity included frequency of severe exacerbations over 12 months, symptoms during the day or night, activity limitation, and frequency of SABA reliever use for symptoms [6, 45]. The meta-analysis suggested that more severe or more difficult-to-control asthma is a significant risk factor for OSA (pooled OR = 4.36, 95%CI 2.49–7.64, *I*^2^ = 74.2%, *P* < 0.001) (Fig. 5A).

Then, we conducted a subgroup analysis based on BMI, the I^2^ for asthmatic patients with mean BMI ≥ 27 dramatically decreased to 0% (pooled OR = 2.51, 95%CI 1.95–3.24, *P* < 0.001). Nevertheless, the subgroup of asthmatic patients with mean BMI < 27 still remained a high heterogeneity, presenting a stronger association with OSA (pooled OR = 12.81, 95%CI 3.2–51.37, *I*^2^ = 68.7%, *P* < 0.001) (Fig. 5B). Characteristics of included studies are shown in Table 4.

### Risk of bias and sensitivity analysis

**Fig. 6:**
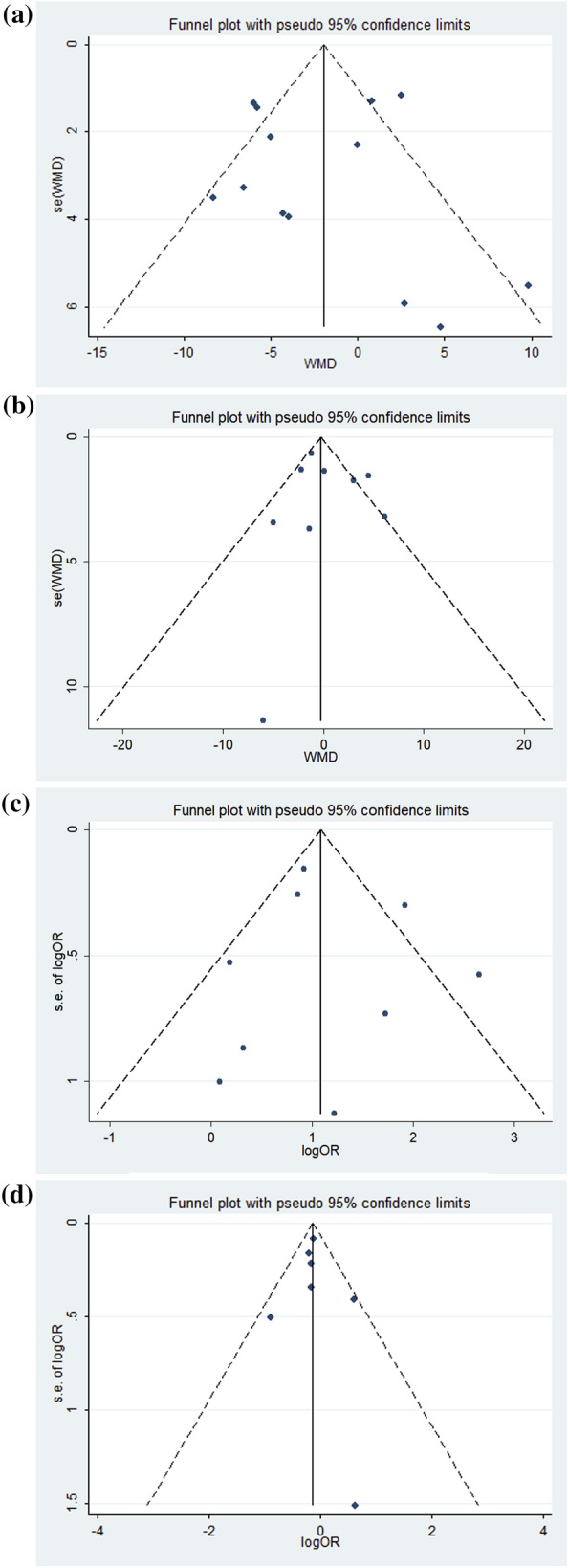
**A** The relationship between OSA and %FEV1 in asthma patients; **B** The relationship between asthma and AHI in OSA patients; **C** The relationship between OSA and asthma severity in asthma patients; **D** The relationship between asthma and OSA severity in asthma patients. The funnel plot analysis of publication bias. OSA: obstructive sleep apnea; %FEV1: forced expiratory volume in one second %predicted; AHI: apnea hypopnea index

AHRQ score for cross-sectional and NOS score for cohort studies are listed in Table 1–4. There were no studies considered low quality according to the criterion and no publication bias was observed in the funnel plot or Egger’s test for (a) the relationship between OSA and %FEV1 in asthmatic patients; (b) the relationship between asthma and AHI in OSA patients; (c) the relationship between OSA and asthma severity in asthmatic patients; and (d) the relationship between asthma and OSA severity in asthmatic patients. (*P* = 0.875, *P* = 0.650, *P* = 0.084, and *P* = 0.535, respectively, Fig. 6, Additional file 1: Fig. S5). The meta-regression revealed that age, gender, and BMI did not affect the statistical significance of the pooled results, showing the association between OSA and more severe or difficult-to-control asthma (*P* = 0.203, *P* = 0.826, *P* = 0.672, respectively). Moreover, sensitivity analyses of two parts in the meta-analysis confirmed that the result was stable with no statistical significance compared with the estimate (Additional file 1: Fig. S6).

## Discussion

To the best of our knowledge, this is the first meta-analysis to investigate the effect of OSA on lung function in asthmatic patients (Part A) and the effect of asthma on PSG in OSA patients (Part B), with a large amount of subjects. The results of Part A suggested that in asthmatic patients complicated with OSA, lung function may be aggravated with decreased %FEV1, especially in children. Nevertheless, the %FEV1 tended to decrease in adult asthma patients complicated with OSA, but did not reach statistical significance. OSA also had a negative effect on asthma symptoms and airway inflammation, as shown by increased ACT and FeNO. OSA did not affect small airway function assessed by FEF_25–75_%. Interestingly, the risk of asthma appeared to be lower in patients with more severe OSA with pooled OR = 0.87. Results of Part B indicated that more severe asthma or more difficult-to-control asthma was associated with OSA with pooled OR = 4.36, independent of age, gender, and BMI. And when OSA patients complicated with asthma, daytime sleepiness was aggravated with increased ESS score, but there was no positive impact on parameters of PSG (AHI, LSpO2, ODI, ArI, and T90%) with extremely low heterogeneity.

### The effect of OSA on asthma

Previous studies have reported that OSA is one of the most important pathophysiological mechanisms related to the worsening of asthma symptoms and control, in addition to shared risk factors and comorbidities [46]. OSA causes chronic systemic inflammation with the activation and release of cytokine and inflammatory mediators such as TNF-a, interleukin-6, vascular endothelial growth factor, pentane, 8-isoprostane, C-reactive protein, leptin and matrix metallopeptidase-9 [47–51]. Many studies have found that OSA could lead to increased sputum neutrophils, which are linked to increased type 1 airway inflammation, airway remodeling, steroid resistance, and increased disease severity in asthma [52, 53]. The meta-analysis indicated that OSA slightly increased FeNO in asthma patients. FeNO is a biomarker widely used to assess airway inflammation and is higher in patients with asthma characterized by type 2 airway inflammation [54]. Nonetheless, the previous study revealed that chronic intermittent hypoxia for OSA reduced type 2 airway inflammation with decreased interleukin-5 and interleukin-13 in an animal model [55]. The measurement of FeNO led to inconsistent results that OSA patients had similar or slightly higher levels of FeNO compared to healthy controls [56]. The specific mechanism and clinical significance of increasing FENO in asthma patients complicated with OSA need to be further explored. In addition, bronchial hyperresponsiveness is closely associated with nocturnal pulmonary blood pooling caused by increasing negative intrathoracic pressures, chronic intermittent hypoxia mediated through vagal neural receptor activation that accompanies the Muller maneuvers of obstructive events and stimulation of the carotid body [1, 36].

%FEV1 and FEV_1_/FVC are the most common spirometric measurements applied to identify both the presence and degree of airflow obstruction [6]. In the subgroup of varying severity of OSA, two included studies [7, 18] showed that patients with asthma and moderate-severe OSA had better lung function (%FEV1, FEV_1_/FVC) than those with AHI < 15/h without heterogeneity. Careful analysis of two studies revealed that baseline %FEV_1_, age, BMI, and gender did not differ between patients with and without OSA. Nonetheless, Oyama and his colleagues showed that 15/20 patients with AHI ≥ 15/h were at asthma treatment stage 4 compared with 4/16 patients with AHI < 15/h [18]. Similarly, another study showed that baseline %FEV_1_ in asthmatic patients with severe OSA was higher than that of patients with moderate OSA (77.1 ± 25.3 vs 65.3 ± 24.6), and medication was similar except for leukotriene antagonist (61.8% vs 30.3%) [12]. When prescribed as monotherapy or as add-on therapy to ICS, leukotriene antagonists can increase FEV_1_ [57]. Therefore, the conflicting results may be because asthma is more severe or difficult to control in the presence of severe OSA, requiring higher levels of asthmatic treatment that presents a better baseline lung function. In addition, OSA was associated with more rapid lung function decline per year in patients with asthma and OSA severity had an essential effect on the rate of decline [12, 58]. Guidelines advise that %FEV_1_ < 60% determines that a patient is at risk of future asthma exacerbations [6]. OSA is strongly connected to asthma exacerbations with decreased lung function.

Although a significant decline in baseline %FEV1 was observed among children asthmatic patients complicated with OSA, it was not seen in adult patients with high heterogeneity. Some studies indicated that airway hyperresponsiveness is a strong risk factor for low lung function, mainly seen in airway parameters (FEV_1_, FEV_1_/FVC) in children [59, 60]. This may be the reason children with asthma and OSA had a greater reduction in lung function compared with adults in our meta-analysis with increasing airway hyperresponsiveness in OSA. According to pathophysiology mechanisms and the result of our study  that %FEV1 tented to decline in adult asthma in OSA, a cohort study controlling many confounding factors such as age, male%, BMI and asthma treatment stage is recommended to investigate the effect of OSA on lung function in asthmatic patients. Blood and urine samples can also be collected for proteomic studies to explore new potential biomarkers, the pathophysiological mechanisms, and the specific differences between children and adults [61]. Poor asthmatic control will slow development and reduce the basal level of future lung function [62–64].

Although more severe OSA aggravated asthma control, the meta-analysis came out that it was not associated with the risk of OSA (OR = 0.87, *P* = 0.047, I2 = 0%). In the two included studies with the largest population (N = 8400), AHI was slightly lower in asthmatic than in non-asthmatic patients with OSA [25, 32] (26.2 vs 23.0 events/h, *P* < 0.001; 12.6 vs 11.3 events/h, *P* < 0.001). In terms of baseline characteristics, those without asthma were more likely to be male and older than those with asthma. Advancing age and male gender will increase the risk and worsen OSA severity [65]. This may suggest that patients with asthma were mainly female and younger, and more likely to appear in the moderate OSA group. Therefore, age and gender were the important confounding factors to account for OR = 0.87, and there is no clear evidence that coexisting with more severe OSA reduces the prevalence of asthma.

Smoking condition is another potential confounding factor of inconsistent lung functions in the adult group. In the study of Wang et al. and Lu et al., the results showed that the group with more smokers had worse average lung function in %FEV_1_ [12, 14]. Smokers with asthma may present an accelerated rate of decline in lung function and may develop persistent airflow obstruction due to airway remodeling, increasing the use of rescue medication and the number of hospitalizations and emergency room visits [66–68]. Additionally, secondhand smoke is an important risk factor for childhood asthma, asthma-like syndrome, and wheezing [69]. Despite tobacco smoking negatively affecting asthma control, OSA is strongly associated with poor control of asthma independent of smoking and other known asthma aggravators [70, 71]. Since the most included original article did not demonstrate detailed information, we failed to undertake a subgroup analysis or meta-regression on smoking conditions. In conclusion, physicians should be alert for the presence of comorbid OSA in patients with asthma, especially those who have low %FEV_1_, preventing accelerated airway remodeling and reduction in lung function.

### The effect of asthma on OSA

The result of Part B in our meta-analysis suggested that more severe asthma or more difficult-to-control asthma was associated with OSA with pooled OR = 4.36. The reasonable explanations may be OSA aggravates asthma, or severe asthma leads to OSA. A large prospective cohort study with 8-year follow-up intervals suggested that patients with asthma experienced a higher risk of OSA than those without asthma (49% vs 28%, *P* < 0.001), and asthma duration was an important predictor of OSA occurrence [72]. Many studies consistently consider that ICS and oral corticosteroids, both common therapies for asthma, also constitute a high risk for OSA occurrence with peri-pharyngeal fat deposition and upper airway myopathy [73, 74]. Asthma may disrupt the balance of forces to maintain pharyngeal airway patency, for example, increased negative intrathoracic pressure may lead to higher pharynx collapsibility during asthma attacks [75, 76]. Collett and colleagues also suggested that asthma could promote a reduction in the surface area of the upper airway with persistent airway mucosal inflammation [77].

Although many studies identified a mechanism by which asthma aggravates OSA, asthma was not obviously associated with increased respiratory events or worsened nocturnal hypoxia (T90% and LSpO_2_) in OSA in our meta-analysis (Table 6). A potential reason for the discrepancy in meta-analysis is that low arousal threshold is more frequent in patients with OSA and co-existing asthma, compared to those who have OSA alone [33]. Asthma is associated with respiratory muscle weakness and greater instability of the respiratory drive, and asthma nocturnal symptoms may result in sleep fragmentation and less total sleep time [78–80]. These pathological mechanisms may contribute to the low arousal threshold in OSA, presented as lower AHI [81]. Sleep structure disorders raised by various asthma severity might account for the inaccurate acquisition of PSG parameters. Most of the original studies in this section did not address the severity of asthma, so we did not perform a subgroup analysis. A recent study suggests that it is more important to consider mean oxygen saturation during sleep when assessing OSA in asthma patients, which is associated with asthma symptoms and lung function [82].

Daytime sleepiness is one of the most important features of OSA evaluated by the ESS score. The results of the meta-analysis indicated that comorbidity with asthma slightly increased ESS in patients with OSA (WMD = 0.60, *P* = 0.007, I^2^ = 0%), which is consistent with the previous study. The risk of daytime sleepiness is 50% higher in asthmatic patients compared to those without asthma, and allergic rhinitis, a common comorbidity of asthma, is independently associated with sleepiness [83]. Impaired sleep quality correlates with worse asthma control and quality of life with daytime sleepiness [84]. Most patients included in studies were suspected or diagnosed with OSA, and sleep physicians may not have investigated patients sufficiently to determine the presence or absence of asthma [39]. We believe that there should be a more pronounced difference in ESS between asthmatics and non-asthmatics and that asthma may have been underdiagnosed on self-report questionnaires due to the similarity of nocturnal asthmatic symptoms to those of OSA.

### Limitations

This study has some important limitations. In the analysis of the relationship between OSA and lung function in asthmatic patients, we failed to lower the high heterogeneity with different subgroup analyses. It is possible that coexisting OSA has a negative effect on asthma with different phenotypes and endotypes. We failed to collect sufficient data on peak expiratory flow, a predictive indicator for exacerbation of asthma. Although comorbidity with asthma did not show a significant effect on PSG in OSA, the included patients with asthma were not grouped according to severity. Whether severe or uncontrolled asthma affects OSA severity requires further study. In the included studies, mismatches in age, gender, BMI, and treatment status between the single disease group and comorbidity group may have contributed to risk bias for meta-analysis, with high heterogeneity in adult patients. OSA and asthma are closely associated with shared comorbidities such as rhinitis, obesity, smoking condition, and gastroesophageal reflux disease, and would have affected our final results [46]. Finally, asthma is a heterogeneous disease (allergic, non-allergic, eosinophilic, neutrophilic, etc.), and the specific relationship between endotypes of asthma and OSA is unknown. Taken together, a large-scale cohort study is needed to investigate the association between OSA and asthma by figuring out the confounding factors derived from phenotypes and endotypes.

## Conclusion

In this meta-analysis, OSA was associated with more severe or more difficult-to-control asthma with decreased %FEV1 and increased airway inflammation, especially in children. %FEV1 tended to decrease in adult asthma patients complicated with OSA, but did not reach statistical significance. The differences between children and adults should be investigated by clinical research and proteomic studies. Asthma increased daytime sleepiness but did not significantly worsen OSA severity despite a lack of a subgroup for severity of asthma. More studies are warranted to investigate the effect of asthma on OSA severity and the impact of different OSA severity on the prevalence of asthma. Overall, clinicians should perform early detection, diagnosis and therapy for OSA in patients with asthma, in order to slow down the rate of airway remodeling and the decline in lung function.

## Supplementary Information

**Additional file 1: Table S1.** Search Medline via PubMed (up to September 2022). **Table S2.** Search Embase via Ovid (up to September 2022). **Table S3.** Search Scopus (up to September 2022). **Table S4.** Subgroup meta-analysis of main outcomes in adults patients with asthma in OSA group and non-OSA group. **Table S5.** Subgroup meta-analysis of main outcomes in adults patients with OSA in asthma group and non-asthma group. **Fig S1. **The meta-analysis of asthma control trial (ACT) in the relationship between OSA and asthma control status. **Fig S2. **The meta-analysis of forced expiratory flow (25–75% of VC) (FEF25–75%) in the relationship between OSA and lung function in asthma patients. **Fig S3. **The meta-analysis of fractional exhaled nitric oxide (FeNO) in asthma patients with or without OSA. **Fig S4. **The meta-analysis of Epworth Sleepiness Scale (ESS) in OSA patients with or without asthma. **Fig S5. **The Egger’s test for publication bias. (a): The relationship between OSA and %FEV1 in asthma patients(b): The relationship between asthma and AHI in OSA patients(c): The relationship between OSA and asthma severity in asthma patients(d): The relationship between asthma and OSA severity in asthma patients.**Fig S6. **The sensitivity analyses for assessing the stability of the results. (a): The relationship between OSA and %FEV1 in asthma patients(b): The relationship between asthma and AHI in OSA patients(c): The relationship between OSA and asthma severity in asthma patients(d): The relationship between asthma and OSA severity in asthma patients.

## Data Availability

The data sets used or analyzed during the current study are available from the corresponding author on request.

## Abbreviations

OSA: Obstructive sleep apnea
ICS: Inhaled corticosteroids
%FEV_1_: Forced expiratory volume in one second %predicted
FVC%: Forced vital capacity % predicted
FEV_1_/FVC: Forced expiratory volume in one second/forced vital capacity
FEF_25-75_%: Forced expiratory flow 25–75% of VC
ACT: Asthma control test
PSG: Polysomnography
AHI: Apnea/hypopnea index
LSpO2: Lowest peripheral oxygen saturation
ODI: Oxygen desaturation index
ArI: Arousal index
ESS: Epworth sleepiness scale
T90%: Percent sleeping time in which oxygen saturation was below 90%
WMD: Weight mean difference
AHRQ: Agency for healthcare research and quality
NOS: Newcastle–Ottawa scale
GINA: The global initiative for asthma
