# Supplementary material for: The relationship between obstructive sleep apnea and asthma severity and vice versa: a systematic review and meta-analysis
Source: Eur J Med Res. 2023 Mar 30;28:139. doi: 10.1186/s40001-023-01097-4 (PMC10062016; doi:10.1186/s40001-023-01097-4)

**The relationship between obstructive sleep apnea and asthma severity and vice versa: a systematic review and meta-analysis**

Donghao Wang^1^, Yanyan Zhou^1^, Riken Chen^1^, Xiangxia Zeng^1^, Sun Zhang^1^, Xiaofen Su^1^, Yateng Luo^1^, Yongkang Tang^1^, Shiwei Li^1^, Zhiyang Zhuang^1^, Dongxing Zhao^1^, Yingying Ren^2^, Nuofu Zhang^1^

Affiliations:

^1^ Sleep Medicine Center, State Key Laboratory of Respiratory Disease, National Clinical Research Center for Respiratory Disease, Guangzhou Institute of Respiratory Health, The First Affiliated Hospital of Guangzhou Medical University, Guangzhou, Guangdong, 510120, People’s Republic of China;

^2^ Guangzhou Medical University, the First Affiliated Hospital of Guangzhou Medical University, Guangzhou, Guangdong, 510120, People’s Republic of China;

**Table leagues**

| Table | Title |
| --- | --- |
| Table S1 | Searching strategy for Medline via PubMed. |
| Table S2 | Searching strategy for Embase via Ovid. |
| Table S3 | Searching strategy for Scopus. |
| Table S4 | Subgroup meta-analysis of main outcomes in adults patients with asthma in OSA group and non-OSA group. |
| Table S5 | Subgroup meta-analysis of main outcomes in adults patients with OSA in asthma group and non-asthma group. |

**Figure leagues**

|  | Title | Caption |
| --- | --- | --- |
| Fig S1 | - | The meta-analysis of asthma control trial (ACT) in the relationship between OSA and asthma control status. |
| Fig S2 | - | The meta-analysis of forced expiratory flow (25–75% of VC) (FEF_25–75_%) in the relationship between OSA and lung function in asthma patients. |
| Fig S3 | - | The meta-analysis of fractional exhaled nitric oxide (FeNO) in asthma patients with or without OSA. |
| Fig S4 | - | The meta-analysis of Epworth Sleepiness Scale (ESS) in OSA patients with or without asthma. |
| Fig S5 | (a): The relationship between OSA and %FEV1 in asthma patients  (b): The relationship between asthma and AHI in OSA patients  (c): The relationship between OSA and asthma severity in asthma patients  (d): The relationship between asthma and OSA severity in asthma patients | The Egger’s test for publication bias. |
| Fig S6 | (a): The relationship between OSA and %FEV1 in asthma patients  (b): The relationship between asthma and AHI in OSA patients  (c): The relationship between OSA and asthma severity in asthma patients  (d): The relationship between asthma and OSA severity in asthma patients | The sensitivity analyses for assessing the stability of the results. |

**Table S1**

Search Medline via PubMed (up to September 2022)

| **#** | **Searches** | **Results** |
| --- | --- | --- |
| 1. | (“Asthma”[MeSH] OR (ASTHMA[Title/Abstract]) OR (Asthmas[Title/Abstract]) OR (Bronchial Asthma[Title/Abstract]) OR (Asthma, Bronchial[Title/Abstract])) | 192,298 |
| 2. | (“Sleep Apnea, Obstructive”[MeSH] OR (Apneas, Obstructive Sleep[Title/Abstract]) OR (Obstructive Sleep Apneas[Title/Abstract]) OR (Sleep Apneas, Obstructive[Title/Abstract]) OR (Obstructive Sleep Apnea Syndrome[Title/Abstract]) OR (Obstructive Sleep Apnea[Title/Abstract]) OR (OSAHS[Title/Abstract]) OR (Syndrome, Sleep Apnea, Obstructive[Title/Abstract]) OR (Sleep Apnea Syndrome, Obstructive[Title/Abstract]) OR (Apnea, Obstructive Sleep[Title/Abstract]) OR (Sleep Apnea Hypopnea Syndrome[Title/Abstract]) OR (Syndrome, Obstructive Sleep Apnea[Title/Abstract]) OR (Upper Airway Resistance Sleep Apnea Syndrome[Title/Abstract]) OR (Syndrome, Upper Airway Resistance, Sleep Apnea[Title/Abstract]) ) | 36,607 |
| 3. | #1 and #2 | 691 |
| 4. | (#3 ) NOT (review[Publication Type]) | 484 |

**Table S2**

Search Embase via Ovid (up to September 2022)

| **#** | **Searches** | **Results** |
| --- | --- | --- |
| 1. | ((Asthma:ti,ab) OR (Asthmas:ti,ab)) | 244,087 |
| 2. | ((Obstructive Sleep Apneas:ti,ab) OR (Obstructive Sleep Apnea:ti,ab) OR (OSA:ti,ab) OR (obstructive sleep apneoa:ti,ab) OR (Sleep Apnea Hypopnea Syndrome:ti,ab) OR (Sleep Apneoa Hypopneoa Syndrome:ti,ab) OR (Upper Airway Resistance Sleep Apnea Syndrome:ti,ab)) | 59,858 |
| 3. | #1 and #2 | 1,504 |
| 4. | #3 AND ([article]/lim OR [article in press]/lim OR [data papers]/lim) | 461 |

**Table S3**

Search Scopus (up to September 2022)

| **#** | **Searches** | **Results** |
| --- | --- | --- |
| 1. | TITLE-ABS-KEY((Asthma) OR (Asthmas)) | 284,718 |
| 2. | TITLE-ABS-KEY ( ( obstructive AND sleep AND apneas ) OR ( obstructive AND sleep AND apnea ) OR ( osa ) OR ( obstructive AND sleep AND apneoa ) OR ( sleep AND apnea AND hypopnea AND syndrome ) OR ( sleep AND apneoa AND hypopneoa AND syndrome ) OR ( upper AND airway AND resistance AND sleep AND apnea AND syndrome ) ) | 94,033 |
| 3. | #1 and #2 | 1,404 |
| 4. | #3 AND ( LIMIT-TO ( DOCTYPE ,  "ar" ) | 794 |

Table S4

Subgroup meta-analysis of main outcomes in adults patients with asthma in OSA group and non-OSA group.

| **Outcomes and subgroups** | **No. of studies** | **Population** | **WMD** | **95%CI** | **I^2^ (%)** | **PH** | **Statistical model** | **PZ** | **Between group P value** |
| --- | --- | --- | --- | --- | --- | --- | --- | --- | --- |
| **FEV1 (%)** |  |  |  |  |  |  |  |  |  |
| All | 9 | 793 | -1.24 | -4.56, 2.08 | 78.1 | < 0.001 | Random | 0.464 | - |
| Severity of OSA |  |  |  |  |  |  |  |  |  |
| AHI ≥ 5/h | 7 | 895 | -2.34 | -5.78, 1.10 | 81.1 | < 0.001 | Random | 0.182 | 0.028 |
| AHI ≥ 15/h | 2 | 98 | 7.69 | -0.54, 15.93 | 0 | 0.557 |  | 0.067 | - |
| Severity of asthma |  |  |  |  |  |  |  |  |  |
| Routine | 6 | 578 | -2.16 | -6.16, 1.83 | 76.9 | 0.001 | Random | 0.289 | 0.273 |
| Uncontrolled | 3 | 215 | -1.14 | -3.22, 5.51 | 33.5 | 0.222 |  | 0.607 | - |
| Age (yr) |  |  |  |  |  |  |  |  |  |
| ≥ 50 | 4 | 289 | -0.89 | -7.89, 5.62 | 68.0 | 0.025 | Random | 0.770 | 0.985 |
| < 50 | 5 | 504 | -0.91 | -4.42, 2.60 | 73.0 | 0.005 |  | 0.611 | - |
| Male (%) |  |  |  |  |  |  |  |  |  |
| ≥ 50 | 4 | 476 | -0.68 | -4.90, 3.53 | 53.7 | 0.090 | Random | 0.751 | 0.788 |
| < 50 | 5 | 317 | -1.63 | -7.06, 3.81 | 86.5 | < 0.001 |  | 0.557 | - |
| BMI (kg/m^2^) |  |  |  |  |  |  |  |  |  |
| ≥ 27 | 3 | 123 | -0.51 | -7.80, 6.78 | 91.4 | < 0.001 | Random | 0.892 | 0.995 |
| < 27 | 6 | 670 | -0.53 | -4.78, 3.71 | 60.9 | 0.037 |  | 0.805 | - |
| **FEV1/FVC (%)** |  |  |  |  |  |  |  |  |  |
| All | 7 | 641 | -0.04 | -2.28, 2.37 | 67.3 | 0.005 | Random | 0.972 | - |
| Severity of OSA |  |  |  |  |  |  |  |  |  |
| AHI ≥ 5/h | 5 | 543 | -0.79 | -3.26, 0.69 | 72.5 | 0.006 | Random | 0.535 | 0.041 |
| AHI ≥ 15/h | 2 | 98 | 4.83 | 0.05, 9.61 | 0 | 0.482 |  | 0.047 | - |
| Severity of asthma |  |  |  |  |  |  |  |  |  |
| Routine | 5 | 547 | -1.19 | -3.93, 3.55 | 76.1 | 0.002 | Random | 0.920 | 0.550 |
| Uncontrolled | 2 | 94 | 1.01 | -0.27, 2.30 | 0 | 0.873 |  | 0.123 | - |
| Age (yr) |  |  |  |  |  |  |  |  |  |
| ≥ 50 | 3 | 137 | 1.23 | -2.56, 4.22 | 68.2 | 0.010 | Random | 0.520 | 0.775 |
| < 50 | 4 | 504 | -0.91 | -3.37, 1.50 | 71.2 | 0.008 |  | 0.452 | - |
| Male (%) |  |  |  |  |  |  |  |  |  |
| ≥ 50 | 4 | 476 | -0.23 | -3.53, 4.07 | 62.3 | 0.047 | Random | 0.907 | 0.788 |
| < 50 | 3 | 165 | 0.48 | -4.10, 5.06 | 79.5 | 0.008 |  | 0.837 | - |
| BMI (kg/m^2^) |  |  |  |  |  |  |  |  |  |
| ≥ 27 | 2 | 93 | 1.01 | -0.27, 2.30 | 0 | 0.873 | Random | 0.123 | 0.816 |
| < 27 | 4 | 498 | -1.00 | -3.14, 5.15 | 74.8 | 0.008 |  | 0.635 | - |

WMD: weighted mean difference. CI: confidence interval. PZ: P value for Z test. PH: P value based on Q test for between-study heterogeneity. %FEV1: forced expiratory volume in one second (%predicted). OSA: obstructive sleep apnea. AHI: apnea/hypopnea index. FVC%: forced vital capacity (%predicted). BMI: body mass index.

Table S5.

Subgroup meta-analysis of main outcomes in adults patients with OSA in asthma group and non-asthma group.

| **Outcomes and subgroups** | **No. of studies** | **Population** | **WMD** | **95%CI** | **I^2^ (%)** | **PH** | **Statistical model** | **PZ** | **Between group P value** |
| --- | --- | --- | --- | --- | --- | --- | --- | --- | --- |
| AHI (events/hour) |  |  |  |  |  |  |  |  |  |
| All | 7 | 9431 | 0.78 | -1.95, 3.38 | 74.2 | 0.001 | Random | 0.559 | - |
| Severity of OSA |  |  |  |  |  |  |  |  |  |
| AHI ≥ 5/h | 4 | 3692 | 0.69 | -2.98, 4.36 | 73.1 | 0.011 | Random | 0.839 | 0.599 |
| AHI ≥ 15/h | 3 | 5739 | 0.63 | -5.45, 6.71 | 82.1 | 0.004 |  | 0.718 | - |
| Age (yr) |  |  |  |  |  |  |  |  |  |
| ≥ 60 | 2 | 2893 | 0.58 | -3.48, 4.65 | 80.2 | 0.024 | Random | 0.774 | 0.977 |
| < 60 | 5 | 6538 | 0.67 | -3.93, 5.28 | 76.2 | 0.002 |  | 0.778 | - |
| Male (%) |  |  |  |  |  |  |  |  |  |
| ≥ 60 | 4 | 6292 | 1.41 | -3.02, 5.85 | 68.7 | 0.022 | Random | 0.532 | 0.987 |
| < 60 | 2 | 2945 | 1.48 | -4.10, 7.05 | 91.3 | 0.001 |  | 0.602 | - |
| BMI (kg/m^2^) |  |  |  |  |  |  |  |  |  |
| ≥ 32 | 4 | 6414 | -0.82 | -5.79, 4.15 | 58.2 | 0.067 | Random | 0.746 | 0.403 |
| < 32 | 3 | 3017 | 1.89 | -0.89, 5.87 | 86.5 | 0.001 |  | 0.351 | - |

WMD: weighted mean difference. CI: confidence interval. PZ: P value for Z test. PH: P value based on Q test for between-study heterogeneity. AHI: apnea/hypopnea index. BMI: body mass index.

Fig S1


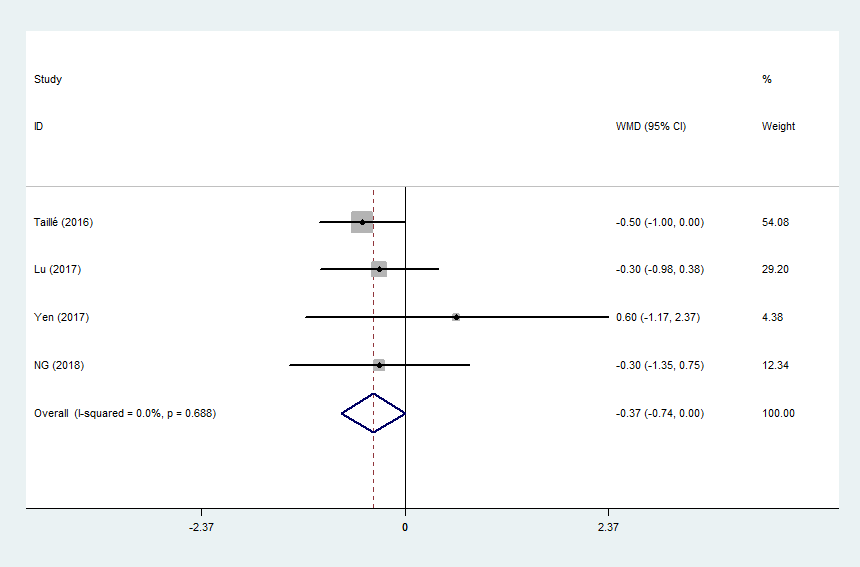


Fig S2


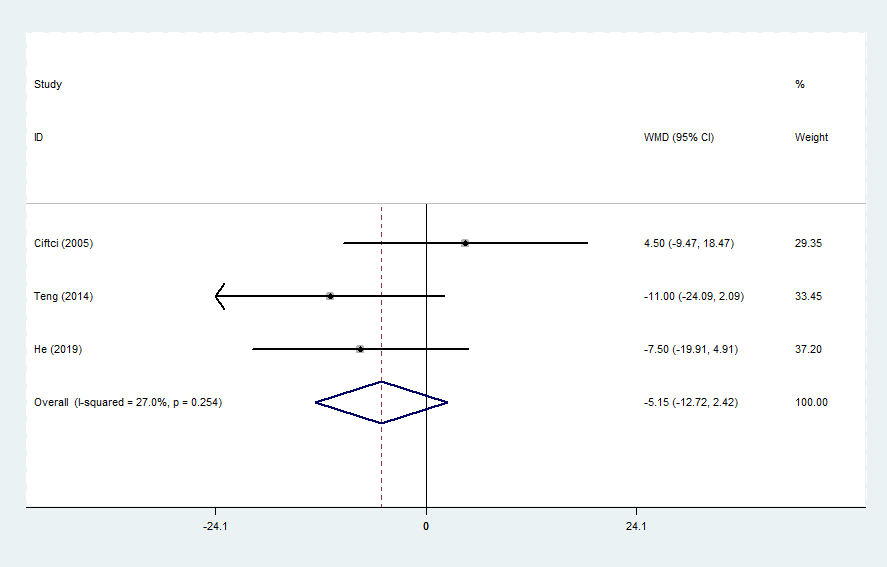


Fig S3


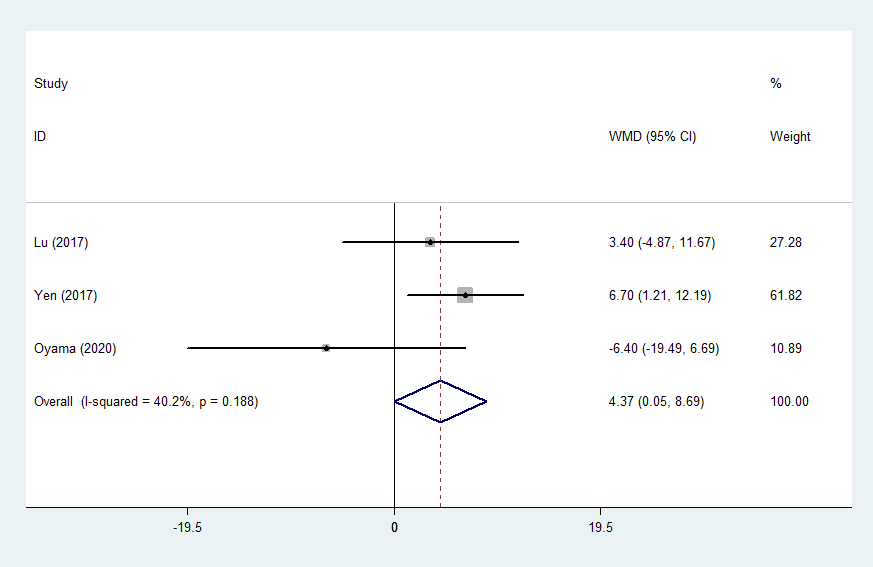


Fig S4


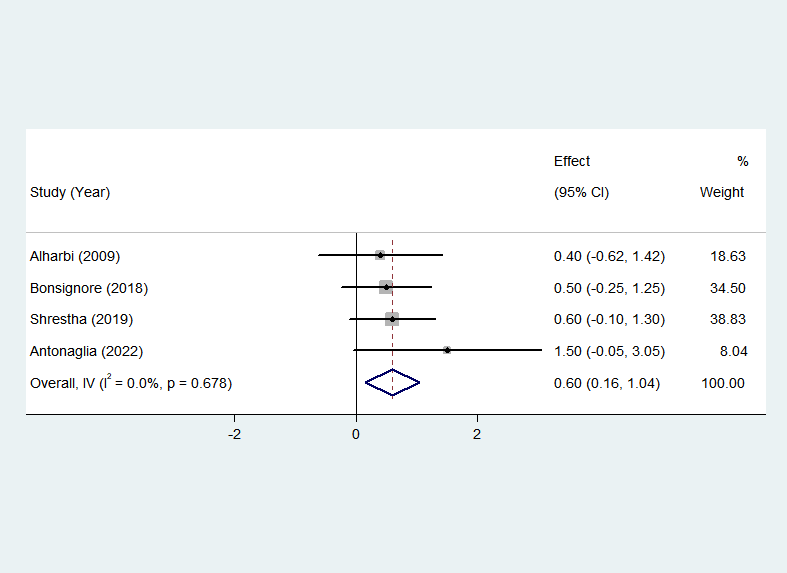


Fig S5

(a)


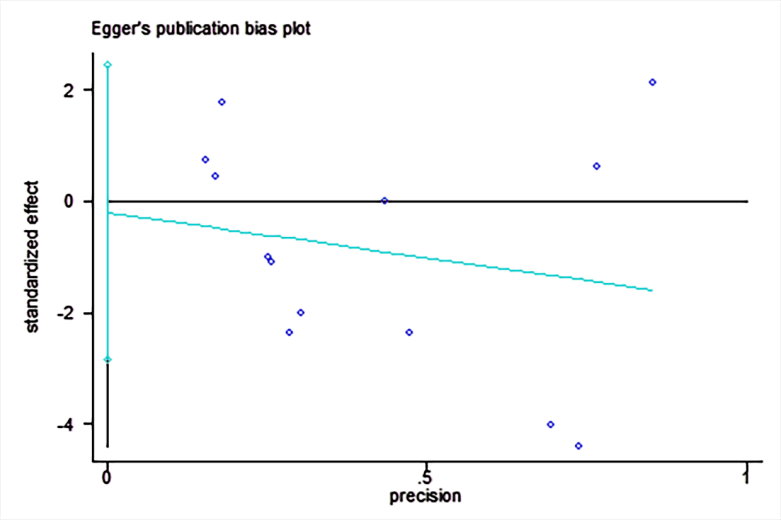


(b)


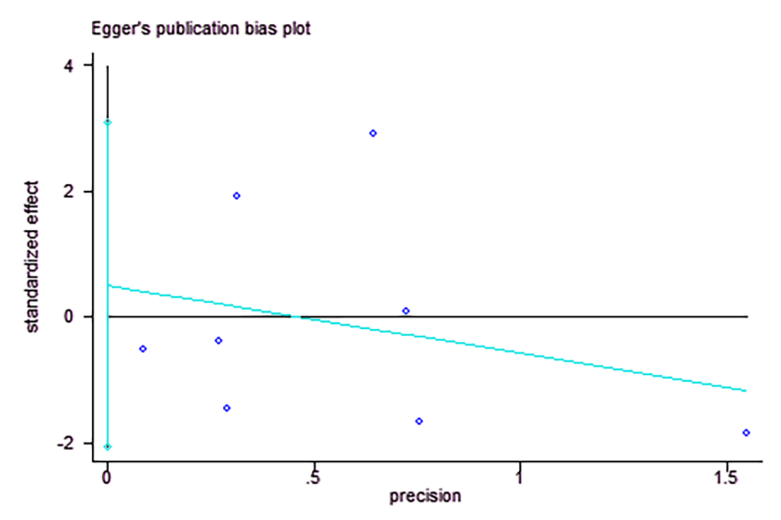


(c)


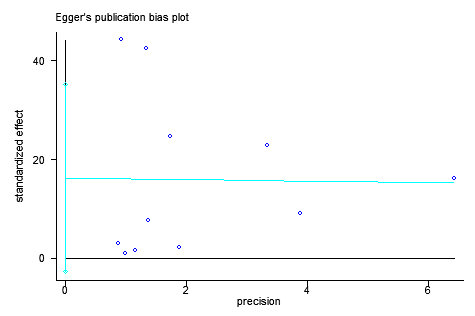


(d)


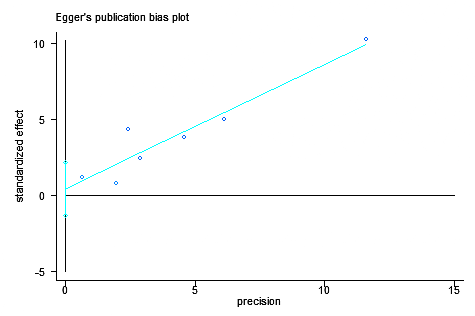


Fig S6

(a)


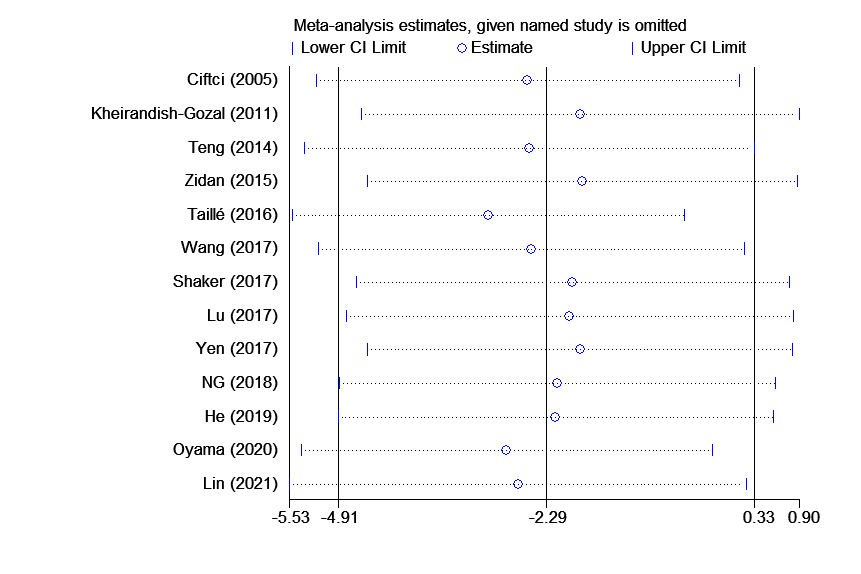


(b)


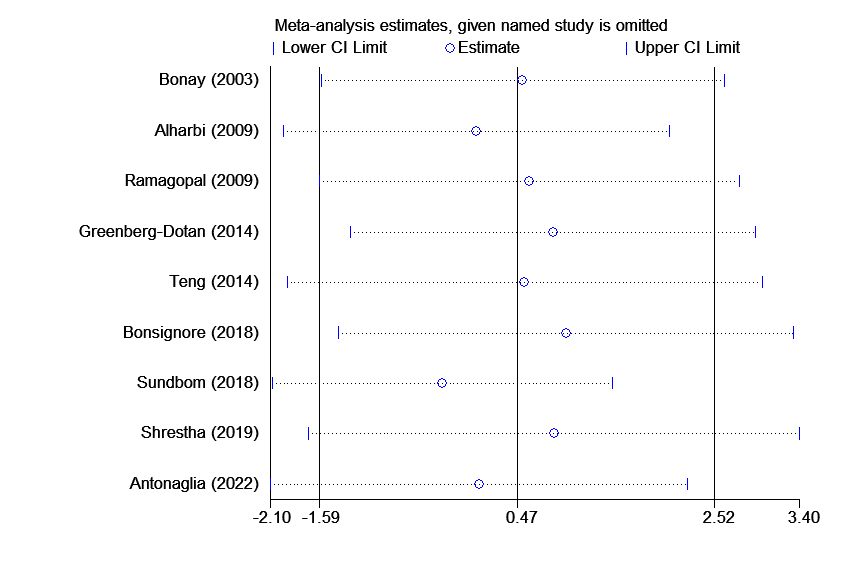


(c)


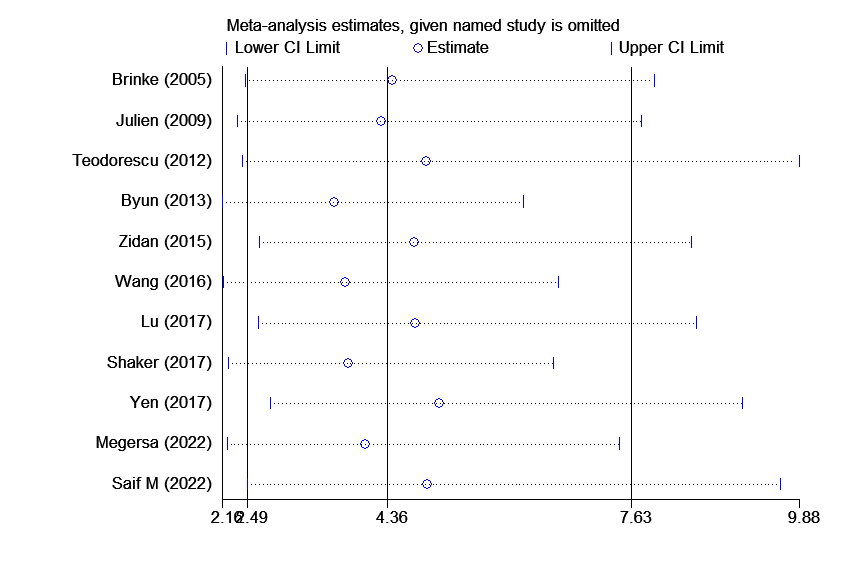


(d)


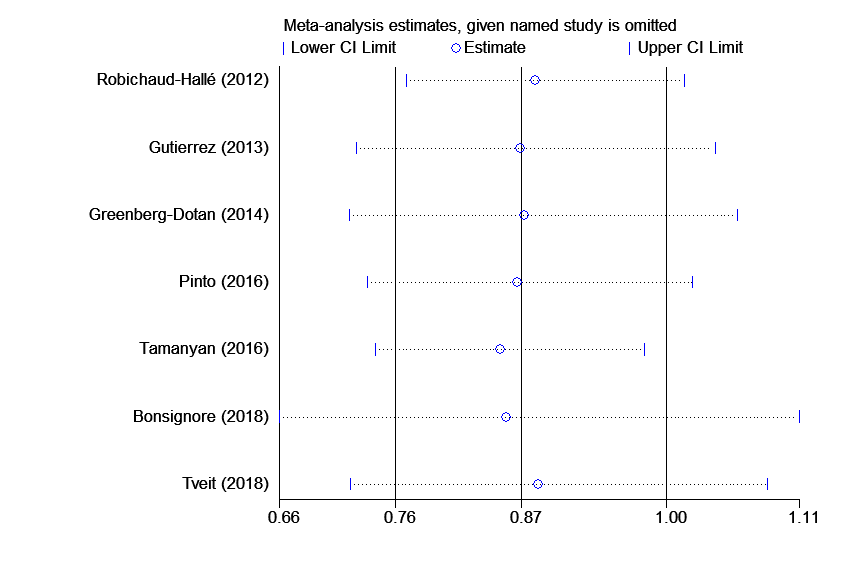

Supplement: Supplementary file 1 — Additional file 1: Table S1. Search Medline via PubMed (up to September 2022). Table S2. Search Embase via Ovid (up to September 2022). Table S3. Search Scopus (up to September 2022). Table S4. Subgroup meta-analysis of main outcomes in adults patients with asthma in OSA group and non-OSA group. Table S5. Subgroup meta-analysis of main outcomes in adults patients with OSA in asthma group and non-asthma group. Fig S1. The meta-analysis of asthma control trial (ACT) in the relationship between OSA and asthma control status. Fig S2. The meta-analysis of forced expiratory flow (25–75% of VC) (FEF25–75%) in the relationship between OSA and lung function in asthma patients. Fig S3. The meta-analysis of fractional exhaled nitric oxide (FeNO) in asthma patients with or without OSA. Fig S4. The meta-analysis of Epworth Sleepiness Scale (ESS) in OSA patients with or without asthma. Fig S5. The Egger’s test for publication bias. (a): The relationship between OSA and %FEV1 in asthma patients(b): The relationship between asthma and AHI in OSA patients(c): The relationship between OSA and asthma severity in asthma patients(d): The relationship between asthma and OSA severity in asthma patients.Fig S6. The sensitivity analyses for assessing the stability of the results. (a): The relationship between OSA and %FEV1 in asthma patients(b): The relationship between asthma and AHI in OSA patients(c): The relationship between OSA and asthma severity in asthma patients(d): The relationship between asthma and OSA severity in asthma patients. [file 40001_2023_1097_MOESM1_ESM.docx]
